# Temporal control in shell–core structured nanofilm for tracheal cartilage regeneration: synergistic optimization of anti-inflammation and chondrogenesis

**DOI:** 10.1093/rb/rbae040

**Published:** 2024-04-11

**Authors:** Wen Zhao, Fanglan Xu, Yumei Shen, Qifeng Ding, Yifei Wang, Leilei Liang, Wufei Dai, Yongbing Chen

**Affiliations:** Department of Thoracic Surgery, The Second Affiliated Hospital of Soochow University, Suzhou, 215004, China; Department of Thoracic Surgery, Tongren Hospital, Shanghai Jiao Tong University, School of Medicine, Shanghai, 200050, China; Department of Thoracic Surgery, The Second Affiliated Hospital of Soochow University, Suzhou, 215004, China; Operation Room Department, The Second Affiliated Hospital of Soochow University, Suzhou, 215004, China; Department of Thoracic Surgery, The Second Affiliated Hospital of Soochow University, Suzhou, 215004, China; Department of Thoracic Surgery, The Second Affiliated Hospital of Soochow University, Suzhou, 215004, China; Department of Gynecologic Oncology, Zhejiang Cancer Hospital, Hangzhou, 310005, China; Department of Plastic and Reconstructive Surgery, Shanghai Ninth People’s Hospital, Shanghai Jiao Tong University, School of Medicine, Shanghai, 200011, China; Department of Thoracic Surgery, The Second Affiliated Hospital of Soochow University, Suzhou, 215004, China

**Keywords:** temporal control, core–shell structured, nanofilm, tracheal cartilage regeneration, anti-inflammation

## Abstract

Cartilage tissue engineering offers hope for tracheal cartilage defect repair. Establishing an anti-inflammatory microenvironment stands as a prerequisite for successful tracheal cartilage restoration, especially in immunocompetent animals. Hence, scaffolds inducing an anti-inflammatory response before chondrogenesis are crucial for effectively addressing tracheal cartilage defects. Herein, we develop a shell–core structured PLGA@ICA-GT@KGN nanofilm using poly(lactic-co-glycolic acid) (PLGA) and icariin (ICA, an anti-inflammatory drug) as the shell layer and gelatin (GT) and kartogenin (KGN, a chondrogenic factor) as the core via coaxial electrospinning technology. The resultant PLGA@ICA-GT@KGN nanofilm exhibited a characteristic fibrous structure and demonstrated high biocompatibility. Notably, it showcased sustained release characteristics, releasing ICA within the initial 0 to 15 days and gradually releasing KGN between 11 and 29 days. Subsequent *in vitro* analysis revealed the potent anti-inflammatory capabilities of the released ICA from the shell layer, while the KGN released from the core layer effectively induced chondrogenic differentiation of bone marrow stem cells (BMSCs). Following this, the synthesized PLGA@ICA-GT@KGN nanofilms were loaded with BMSCs and stacked layer by layer, adhering to a ‘sandwich model’ to form a composite sandwich construct. This construct was then utilized to repair circular tracheal defects in a rabbit model. The sequential release of ICA and KGN facilitated by the PLGA@ICA-GT@KGN nanofilm established an anti-inflammatory microenvironment before initiating chondrogenic induction, leading to effective tracheal cartilage restoration. This study underscores the significance of shell–core structured nanofilms in temporally regulating anti-inflammation and chondrogenesis. This approach offers a novel perspective for addressing tracheal cartilage defects, potentially revolutionizing their treatment methodologies.

## Graphical abstract

**Figure:**
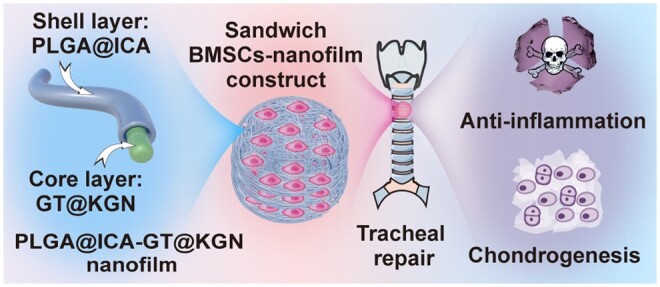

## Introduction

Repairing tracheal cartilage defects presents a significant challenge in thoracic surgery. Firstly, ensuring unobstructed airways and avoiding postoperative complications such as airway stenosis or obstruction due to repair procedures poses considerable surgical difficulty [1]. Secondly, patients undergoing tracheal cartilage defect repair surgeries face risks of infection and complications [2]. Therefore, there is an urgent need to develop a new therapeutic strategy to address these issues. The continuous development of cartilage tissue engineering technology brings new hope for the treatment of tracheal cartilage injuries [3–5].

While satisfactory engineered cartilage substitutes have been generated *in vitro*, robust immune responses often lead to repair failure once transplanted into immunocompetent animal models [6]. Abundant evidence shows that unavoidable inflammatory reactions *in vivo* have multifaceted negative effects on cartilage regeneration. Firstly, inflammatory cytokines (such as interleukin-1β (IL-1β) and tumor necrosis factor-α (TNF-α)) cause damage and apoptosis of chondrocytes [7]. Secondly, in the inflammatory environment, inflammatory factors inhibit the activity of chondrocytes in synthesizing extracellular matrix (ECM) molecules such as collagen and proteoglycans, thereby affecting the synthesis and repair of cartilage tissue [8]. Additionally, under inflammatory conditions, the activity of endothelial cells and fibroblasts is suppressed, hindering new blood vessel growth and leading to insufficient nutrient supply to cartilage tissue, thereby affecting cartilage regeneration and repair processes [9, 10]. Finally, inflammation often leads to the formation of fibrous tissue, which is usually harder in texture, functionally inferior and unfavorable for normal cartilage function recovery [11]. Since the trachea is directly exposed to the external environment, it is more susceptible to the effects of inflammation, leading to repair failure. Therefore, addressing inflammatory reactions during tracheal cartilage repair is the primary focus of using cartilage tissue engineering to solve tracheal cartilage repair problems.

However, with the continuous development of tissue engineering research, an increasing number of studies have found that relying solely on anti-inflammatory measures without chondrogenic induction may result in poor quality of newly formed cartilage, prolonged cartilage regeneration processes and incomplete cartilage repair [12]. Therefore, achieving efficient chondrogenic induction after establishing an anti-inflammatory microenvironment has become a key focus. Consequently, there is an urgent need to develop a staged system that can first establish an anti-inflammatory microenvironment *in vivo* and then achieve chondrogenic induction in a temporally regulated manner.

Electrospinning, as a classic method for biological regeneration materials, plays a significant role in tissue engineering due to its ability to mimic the structure of the ECM and its excellent physical and chemical properties [13]. For instance, Peredo *et al*. improved goat intervertebral disc herniation by loading anti-inflammatory drugs onto electrospun membranes [14]. Furthermore, the development of coaxial electrospinning technology has enabled the creation of multifunctional nanofilms with timed-release core–shell structures [15]. The core–shell structured nanofilms offer several advantages. Firstly, the core–shell architecture allows for the encapsulation of drugs within the core, while the shell serves to delay and regulate drug release, enabling precise control over drug loading, release rate and duration, thereby meeting the requirements of temporal regulation [16]. Secondly, by selecting suitable materials and adjusting fabrication processes, precise control over the degradation rate of the nanofilm can be achieved, facilitating sustained and stable drug release and enhancing therapeutic efficacy [17]. For example, He *et al*. used coaxial electrospinning technology to prepare a dual-layer scaffold with timed-release effects, effectively regenerating osteochondral tissue after tumor eradication [18]. Therefore, preparing a nanofilm with a core–shell structure to establish an anti-inflammatory microenvironment *in vivo* first, followed by achieving chondrogenic induction, is necessary.

The selection of a suitable anti-inflammatory drug and its carrier for the shell layer is crucial for establishing an anti-inflammatory microenvironment. Icariin (ICA), a flavonoid compound derived from the natural plant Epimedium, exhibits good safety and tolerability [19]. Additionally, ICA can inhibit the production of inflammatory mediators and the activation of inflammatory cells, and it possesses antioxidative and immunomodulatory effects, effectively alleviating inflammatory reactions [20]. Poly(lactic-co-glycolic acid) (PLGA) is a biodegradable polymer widely used as a classic tissue engineering scaffold. PLGA demonstrates excellent biocompatibility, and the release efficiency of drugs can be controlled by adjusting the ratio of lactide and glycolide monomers [21]. Therefore, PLGA in combination with ICA logically serves as the shell layer for the anti-inflammatory effect.

Secondly, the composition of the core layer significantly influences tracheal cartilage regeneration. Kartogenin (KGN) is widely applied in regeneration to promote cartilage formation, collagen synthesis, and regulate the development of cartilage tissue. Studies have shown its potential to promote cartilage regeneration by stimulating the differentiation of endogenous bone marrow stem cells (BMSCs) [22]. Gelatin (GT), derived from collagen, is considered an ideal carrier for drugs due to its biocompatibility, degradability and solubility in various solvents [23]. Therefore, KGN-loaded GT could be used as the core layer for chondrogenic induction.

**Scheme 1.**
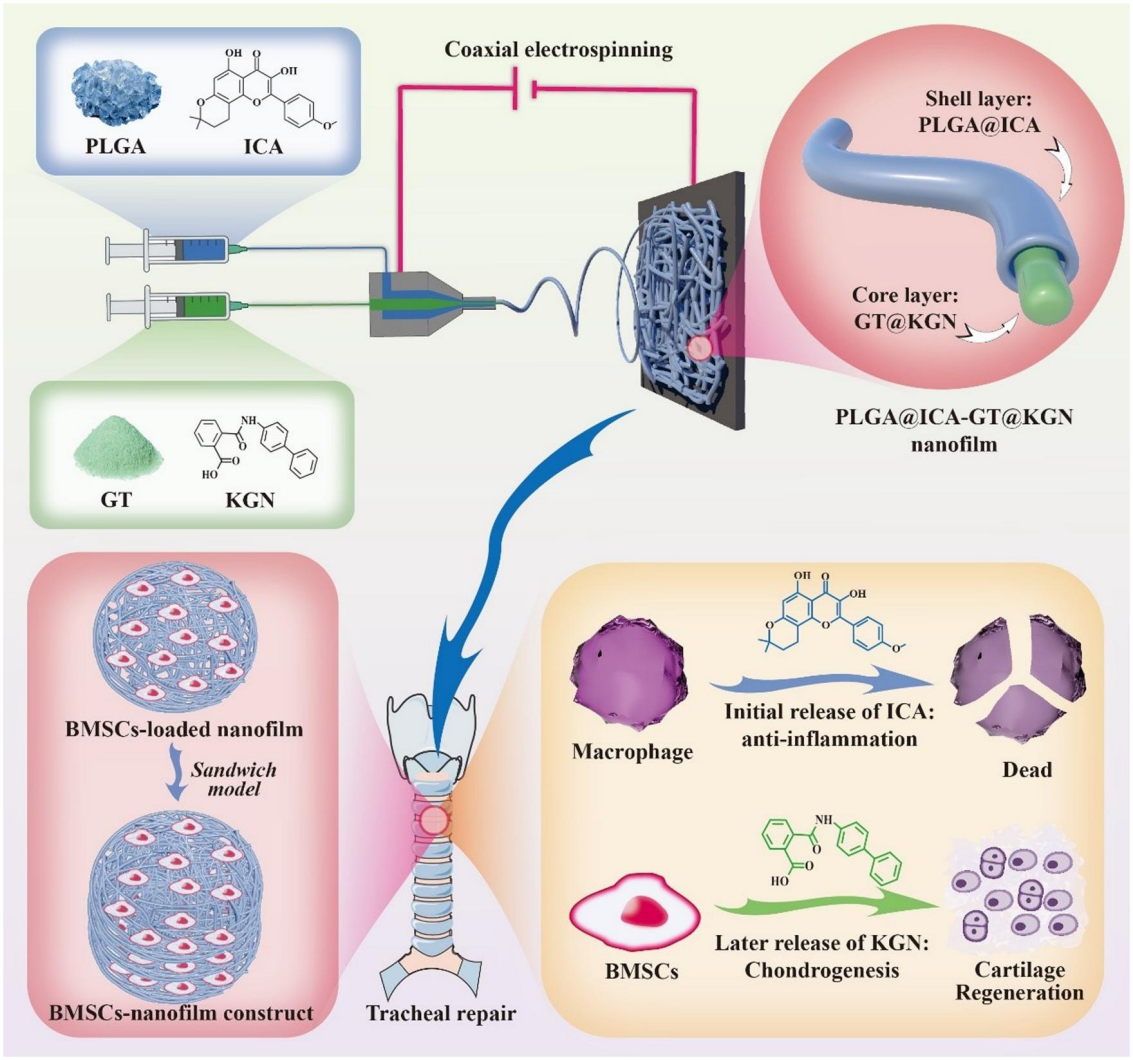
Overall study design. A spinning solution composed of PLGA and ICA formed the shell layer, while another spinning solution comprised of GT and KGN formed the core layer. These solutions underwent coaxial electrospinning to produce a core–shell structured PLGA@ICA-GT@KGN nanofilm. Subsequently, BMSCs were loaded onto the synthesized nanofilms and layered using a ‘sandwich model’ to create a sandwich construct. This construct was then utilized for repairing circular tracheal defects. The rationale behind this design lies in the initial release of ICA from the shell layer, aiming to impart anti-inflammatory effects. Subsequent release of KGN from the core layer is intended to promote chondrogenesis of BMSCs, thereby enhancing cartilage regeneration for the restoration of the trachea.

In summary, this study aims to prepare a core–shell structured nanofilm named PLGA@ICA-Gel@KGN using coaxial electrospinning technology. The shell layer of this nanofilm consists of PLGA and ICA, while the core layer comprises GT and KGN. BMSCs are used as seed cells and layered with the nanofilm using a ‘sandwich model’ for repairing circular tracheal cartilage defects (Scheme 1). The shell layer first releases ICA to establish an anti-inflammatory microenvironment, followed by the release of KGN to promote BMSCs' chondrogenesis, thus achieving efficient repair of tracheal cartilage injuries. To validate its efficacy, the physicochemical properties of the PLGA@ICA-Gel@KGN nanofilm were first evaluated. Subsequently, its biocompatibility, *in vitro* anti-inflammatory and cartilage regeneration effects were assessed. Finally, the staged anti-inflammatory and chondrogenic effect of this nanofilm in repairing tracheal cartilage defects was verified in a rabbit tracheal cartilage defect model.

## Methods and materials

### Preparation of shell–core structured PLGA@Ica-GT@KGN nanofilm

GT (Sigma-Aldrich, USA) and KGN (Sigma-Aldrich, USA) were dissolved in hexafluoroisopropanol. The resulting GT@KGN solution contained GT at a mass/volume ratio of 12% (w/v) and a KGN concentration of 50 μM. PLA was dissolved in hexafluoroisopropanol at an 8% (w/v) mass/volume ratio. The core solution was prepared by mixing the GT@KGN solution and PLA solution at a volume ratio of 10:3.

The shell solution of PLGA@ICA was formed by dissolving PLGA (5% w/v, Sigma-Aldrich, USA) and ICA (10 μM, Sigma-Aldrich, USA). The core and shell solutions were separately fed into a coaxial spinneret (inner bore diameter: 1 mm; outer bore diameter: 2.2 mm) clamped at the tip with a high-voltage power source. A collection plate was positioned 20 cm away from the voltage source. During electrospinning, the GT@KGN core solution and the PLGA@ICA shell solution advanced at a rate of 0.5 ml/h each.

After spinning, the PLGA@ICA-GT@KGN nanofilm was soaked in a cross-linking solution of 1-(3-Dimethylaminopropyl)-3-ethylcarbodiimide hydrochloride (EDC, Sigma-Aldrich, USA)/*N*-Hydroxysuccinimide (NHS, Sigma-Aldrich, USA) for 12 h. It was then washed with deionized water and subjected to freeze-drying. The EDC/NHS cross-linking solution was prepared by dissolving 0.5 g EDC and 0.3 g NHS in 100 ml of 95% ethanol. Similar methods were employed to prepare PLGA-GT, PLGA@ICA-GT and PLGA-GT@KGN nanofilms.

### Structural and performance characterizations

Initially, the nanofilms were photographed using a single-lens reflex camera for macroscopic observation. Subsequently, PLGA-GT and PLGA@ICA-GT@KGN nanofilms underwent vacuum gold-sputtering treatment and were examined under a scanning electron microscope (SEM, S3400, Hitachi, Japan) at an acceleration voltage of 10 kV for microstructural inspection [24]. The average diameters of PLGA-GT and PLGA@ICA-GT@KGN nanofilms were calculated by randomly selecting 100 nanofibers from the obtained SEM images for analysis using ImageJ software [25].

Further characterization involved subjecting PLGA-GT and PLGA@ICA-GT@KGN nanofilms to transmission electron microscopy (TEM, JEM-2100, JEOL, Japan) by placing a carbon-coated copper mesh close to the collector. This assessment aimed to evaluate the shell–core structures of individual nanofibers [26].

Fourier transform infrared spectroscopy (FTIR) spectra were obtained within the 400–4000 cm^−1^ range using an FTIR spectrometer (Thermo Scientific Nicolet iS20, USA) to analyze PLGA, ICA, GT, KGN and PLGA@ICA-GT@KGN samples.

To determine the *in vitro* degradation rate of PLGA-GT and PLGA@ICA-GT@KGN nanofilms, the samples were dried using a vacuum oven for 2 days. They were then weighed at the same initial mass (*W*_0_) and immersed in 10 ml of PBS (pH = 7.4) at 37°C for 29 days. The samples were retrieved every 2 days, lyophilized and re-weighed as *W*_t_. The degradation rate was calculated using the formula: Degradation rate = (*W*_0_ − *W*_t_)/*W*_0_ × 100%.

Additionally, the release rates of ICA and KGN were determined by immersing 30 mg of PLGA@ICA-GT@KGN nanofilm in 10 ml of PBS buffer at pH = 7.4 and 37°C over 0–29 days. The supernatant was measured every 2 days using a UV spectrophotometer.

### Culture and identification of BMSCs

A male New Zealand white rabbit, 4 weeks old and weighing 2.0–2.5 kg, was obtained from Shanghai Yunde Experimental Farm. Animal experiments adhered to the ‘Guidelines for the Care and Use of Laboratory Animals’ and received approval from the Ethics Committee of Zhejiang Cancer Hospital (2024-02-073). Bone marrow was aspirated from anesthetized rabbits' iliac bones, diluted in heparin-treated centrifuge tubes, and centrifuged at 2000 rpm for 8 min in Dulbecco's Modified Eagle Medium (DMEM, Sigma-Aldrich, USA). The harvested bone marrow particles were cultured in DMEM with 10% fetal bovine serum (FBS, Sigma-Aldrich, USA) and 1% antibiotics (Sigma-Aldrich, USA) for 5 days. The medium was changed to eliminate impurities and unattached cells. BMSCs were cultured at 37°C, 5% CO_2_ and harvested after two passages (P2) for subsequent use.

The P2 BMSCs were collected and subjected to immunofluorescence staining to detect the expression of antigens such as CD44, CD90 and CD34, following the instructions of the immunofluorescence detection kit (Beijing Baiao Leibo Technology Co., Ltd, China).

### Isolation and culture of chondrocytes

For the isolation and culture of chondrocytes, auricular cartilage was harvested from the aforementioned rabbits, immersed in an antibiotic solution for 30 min, minced into approximately 0.5–2.0 mm^3^ pieces, and digested with 0.15% type II collagenase (Sigma, USA) in DMEM for 8 h at 37°C to isolate chondrocytes. The isolated chondrocytes were cultured in DMEM supplemented with 10% FBS and 1% antibiotics at 37°C in 5% CO_2_. Chondrocytes were collected at the P2 for use.

### Biocompatibility assessment

To assess the biocompatibility of the PLGA@ICA-GT@KGN nanofilm, BMSCs and chondrocytes suspensions (5 × 10^5^ cells/ml) were implanted separately into culture dishes (serving as a control), PLGA-GT, PLGA@ICA-GT, PLGA-GT@KGN and PLGA@ICA-GT@KGN groups. The BMSCs and chondrocytes-loaded nanofilm were cultured in DMEM supplemented with 10% FBS and 1% antibiotics, with the medium changed every other day.

After 1 and 5 days of *in vitro* culture, the Live/Dead Cell Viability assay (Invitrogen, USA) was conducted per the manufacturer's instructions [27]. The samples were observed using a laser confocal microscope (Leica Thunder imager 3D, Germany) to assess BMSC and chondrocyte viability within the scaffold. ImageJ software was used for quantitative analysis of cell viability from live/dead staining images.

Furthermore, to observe the cytoskeleton of BMSCs on the nanofilm, F-actin and nuclei were stained with phalloidin (Yeasen, China) and DAPI (Yeasen, China), respectively, and observed using laser confocal microscopy. The proliferation of BMSCs and chondrocyte were quantitatively evaluated on Days 1 and 5 using a Cell Counting Kit-8 (CCK-8, Dojindo, Japan) by measuring absorbance at 450 nm with a spectrophotometer, following the manufacturer's protocol.

### *In vitro* anti-inflammatory performance evaluation

RAW264.7 macrophages obtained from Sunncell Biotechnology Co., Ltd (China) were induced with 1 μg/ml lipopolysaccharide (LPS, Sigma-Aldrich, USA) for 24 h to induce inflammation. These induced RAW264.7 macrophages were separately seeded onto culture dishes (serving as a control), PLGA-GT, PLGA@ICA-GT, PLGA-GT@KGN and PLGA@ICA-GT@KGN nanofilms. The groups were cultured in RPMI 1640 medium containing 10% FBS and 1% antibiotics at 37°C with 5% CO_2_.

After 24 h, macrophages from each group were collected. The expression levels of inflammatory factors, including IL-1β, IL-6 and TNF-α, were examined using western blot (WB) analysis and quantitative real-time polymerase chain reaction (qRT-PCR).

WB analysis was employed to detect the expression levels of inflammatory-related proteins (IL-1β, IL-6 and TNF-α) in RAW264.7 macrophages. Total protein from two scaffold BMSCs groups was extracted and subjected to SDS-PAGE electrophoresis. The protein bands were observed using a WB imaging system [28].

Additionally, the expression levels of inflammatory-related genes (*IL-1β*, *IL-6* and *TNF-α*) were evaluated using qRT-PCR [29]. Total RNA was extracted from chondrocytes using TRIzol (Invitrogen, USA) and reverse-transcribed into cDNA. The qRT-PCR was performed using UltraSYBR Mixture (CWBIO, China), with GAPDH as an internal control. Primer sequences used for qRT-PCR were listed in Table 1.

**Table 1.** The primer sequences used for qRT-PCR

Gene	Forward (5′-3′)	Reverse (5′-3′)
*IL-1β*	AGCCTGACCTTGCAGTGCACC	TGCTCCAGCACTCAGCGTCAC
*IL-6*	AGACAGCCACTCACCTCTTCAG	TTCTGCCAGTGCCTCTTTGCTG
*TNF-α*	AGAACAGCAACTCCAGAACACCCT	TGCCAGTTCCACATCTCGGATCAT
*SOX*9	GCGTCAACGGCTCCAGCAAGA	GCGTTGTGCAGGTGCGGGTAC
*ACAN*	CAGGCTATGAGCAGTGTGATGC	GCTGCTGTCTTTGTCACCCACA
*Col2a1*	CACGCTCAAGTCCCTCAACA	TCTATCCAGTAGTCACCGCTCT
GAPDH	TTGTCGCCATCAATGATCCAT	GATGACCAGCTTCCCGTTCTC

Additionally, immunofluorescence staining was used to determine the expression of IL-6 and TNF-α in RAW264.7 macrophages in the control, PLGA-GT, PLGA@ICA-GT, PLGA-GT@KGN and PLGA@ICA-GT@KGN nanofilm groups after 24 h.

### *In vitro* evaluation of chondrogenic promotion effect

The PLGA-GT, PLGA@ICA-GT, PLGA-GT@KGN and PLGA@ICA-GT@KGN nanofilms were initially incubated in PBS for 14 days to promote KGN release. Following this, BMSCs suspensions (5.0 × 10^4^ cells/ml) were implanted onto these pre-treated nanofilms and culture dishes (used as a control). All BMSCs-loaded nanofilms were cultured in DMEM supplemented with 10% FBS and 1% antibiotics for 14 days. Samples were collected to assess *in vitro* chondrogenic differentiation.

The collected samples were fixed in 4% paraformaldehyde and processed for immunofluorescence staining to analyze the expression of chondrogenic-related proteins, including type II collagen (COL II) and recombinant sex-determining region Y box protein 9 (SOX9). Additionally, qRT-PCR was used to detect the expression levels of cartilage-related genes (*SOX9*, *ACAN* and *Col2a1*). The primer sequences used for qRT-PCR were listed in Table 1.

Furthermore, GAG content was quantified using the Alcian blue method, COL II content was measured via enzyme-linked immunosorbent assay, and total collagen content was assessed based on the hydroxyproline assay [30].

### Development of BMSCs-nanofilm construct using ‘sandwich model’

The PLGA-GT, PLGA@ICA-GT, PLGA-GT@KGN and PLGA@ICA-GT@KGN nanofilms were processed into circular membranes of 5 mm diameter using a 5 mm round punch and sterilized with ethylene oxide. A BMSCs-nanofilm construct was developed based on the established ‘Sandwich Model’ [31].

In brief, P2 BMSCs suspension at a density of 10 × 10^7^ cells/ml was seeded onto the nanofilms to achieve complete saturation. The BMSCs-loaded nanofilms were then stacked layer by layer, totaling four layers, to obtain the BMSCs-nanofilm construct. This construct was statically incubated at 37°C with 5% CO_2_ for 4 h. Subsequently, low glucose DMEM containing 10% FBS was gently added to cover the construct, and cultivation continued for 14 days, with the medium changed every other day.

### *In vivo* anti-inflammatory and chondrogenic performance

Twenty-four male New Zealand white rabbits were randomly divided into four groups: PLGA-GT, PLGA@ICA-GT, PLGA-GT@KGN and PLGA@ICA-GT@KGN. After anesthesia, a surgical incision was made in the neck area of these rabbits to expose the trachea. A circular defect with a diameter of 5 mm was created in the cervical trachea of each rabbit. The BMSCs-nanofilm constructs were prepared as described in ‘*In vitro* evaluation of chondrogenic promotion effect’ section and implanted into the tracheal defect of the host rabbit, then sutured with 5-0 sutures.

After awakening, the rabbits were carefully fed for 8 weeks. The survival rate and healing condition in all four groups were recorded. At 2 weeks, the experimental rabbits underwent tracheoscopy under anesthesia. Thereafter, the rabbits were euthanized for gross observation using a single-lens reflex camera. Additionally, to evaluate the regeneration of tracheal cartilage in each group, the repaired tracheal grafts were carefully dissected and subjected to micro-computed tomography (MicroCT).

The obtained tissues at 2 and 4 weeks were fixed and processed for immunofluorescence CD3 and CD68 staining to analyze inflammation. ImageJ software was used to quantify the fluorescence-positive area. Additionally, hematoxylin–eosin (H&E), Safranin-O and immunohistochemical COL II staining were performed to evaluate cartilage lacunae, cartilage-specific ECM and type II collagen deposition, respectively. GAG and COL II contents were assessed as described in ‘*In vitro* anti-inflammatory performance evaluation’ section.

### Statistical analysis

Statistical analysis was conducted using GraphPad Prism 8.0 software. All quantitative data were from at least three repeated experiments and presented as mean ± standard deviation. One-way analysis of variance (ANOVA) was utilized to assess the statistical significance between different groups. Differences with *P* < 0.05 were considered statistically significant.

## Results

### Preparation and characterization of PLGA@Ica-GT@KGN nanofilm

**Figure 1.**
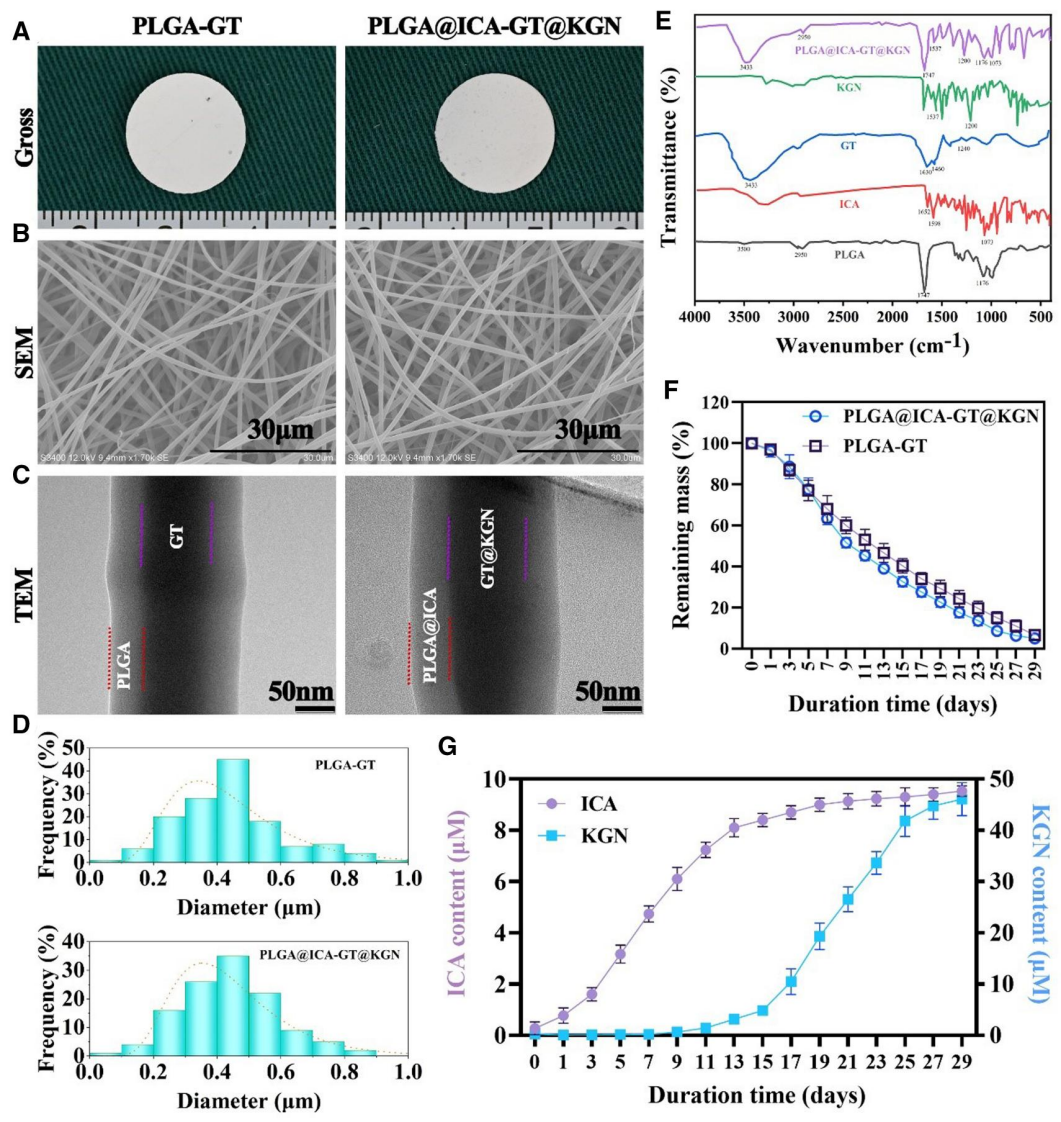
Preparation and characterization of the shell–core structured PLGA@ICA-GT@KGN nanofilm. (**A**) Gross observation showcases the PLGA-GT and PLGA@ICA-GT@KGN nanofilms. (**B**) SEM images depict the differences between PLGA-GT and PLGA@ICA-GT@KGN nanofilms. (**C**) TEM images display the shell–core structure of both PLGA-GT and PLGA@ICA-GT@KGN nanofilms. (**D**) The diameter distribution of both PLGA-GT and PLGA@ICA-GT@KGN nanofilms is depicted. (**E**) FTIR spectra showcase the distinct signatures of PLGA, ICA, GT, KGN and their combined form in the PLGA@ICA-GT@KGN groups. (**F**) The *in vitro* degradation rate of the PLGA@ICA-GT@KGN nanofilm is examined during immersion in PBS (pH = 7.4) at 37°C over 29 days. (**G**) *In vitro* release kinetics of ICA and KGN from the PLGA@ICA-GT@KGN nanofilm are observed during immersion in PBS (pH = 7.4) at 37°C for 29 days.

The PLGA and PLGA@ICA-GT@KGN nanofilms, obtained via coaxial electrospinning, exhibited a strikingly similar white appearance (Figure 1A). SEM analysis confirmed the nano-scaled fibrous structure's uniformity in both PLGA and PLGA@ICA-GT@KGN nanofilms (Figure 1B). TEM images revealed a distinctive shell–core structure in both groups (Figure 1C). Quantitative diameter distribution analysis indicated a similar range of diameter frequencies, spanning 0.1–1.0 μm and predominantly concentrated between 0.3 and 0.5 μm for both PLGA and PLGA@ICA-GT@KGN groups (Figure 1D).

FTIR spectra analysis displayed distinctive peaks for different components. In the PLGA spectrum, characteristic vibrations included OH stretching (3200–3500 cm^−1^), -CH, -CH_2_, -CH_3_ stretching (2950 cm^−1^), carbonyl C=O stretching (1747 cm^−1^) and C–O stretching (1176 cm^−1^). ICA exhibited major peaks at 1652 cm^−1^ (amide I, C=O stretching), 1598 cm^−1^ (amide II, C–N stretching) and 1073 cm^−1^ (C–O–C stretching). GT's spectrum displayed a peak at 3433 cm^−1^, attributed to hydrogen-bonded water and amide-A, along with other peaks for different amide stretches. KGN's spectrum featured a peak at 1,200 cm^−1^ (C-O stretching) and peaks between 1537 and 1596 cm^−1^ (C=C bending of KGN's aromatic ring). In the PLGA@ICA-GT@KGN spectrum, distinctive peaks corresponding to each component validated successful integration.

*In vitro* degradation assays indicated a comparable remaining mass for both PLGA and PLGA@ICA-GT@KGN nanofilms during 0–29 days of incubation (Figure 1F). Release profile analysis revealed the initial release of ICA from the shell layer within the first 15 days, followed by the subsequent release of KGN from the core layer between Days 11 and 29 (Figure 1G). Both compounds exhibited sustained and time-dependent release patterns. The majority of ICA degraded within the initial 15 days, while KGN degradation occurred predominantly between days 13 and 27.

### Biocompatibility assessment

Assessing the survival and proliferation capacity of BMSCs within the PLGA@ICA-GT@KGN nanofilm was pivotal. In this study, we initially identified the BMSCs from the rabbits. The results of immunofluorescence staining confirmed that the obtained BMSCs expressed only CD44 and CD90, while the hematopoietic stem cell marker antigen CD34 showed negative expression (Supplemental Figure S1).

**Figure 2.**
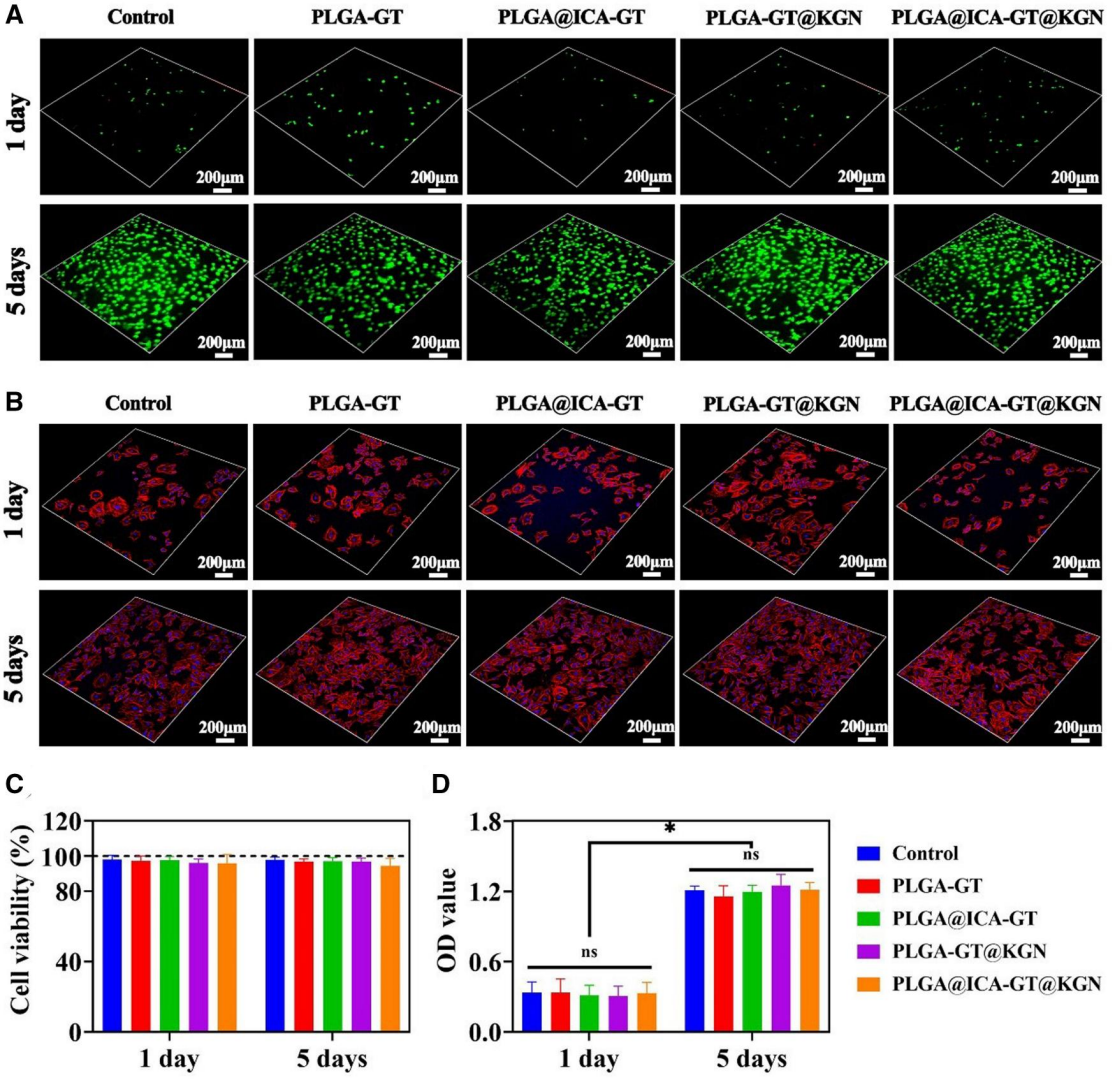
*In vitro* biocompatibility assessment of the PLGA@ICA-GT@KGN nanofilm on BMSCs. (**A**) Live/dead staining and (**B**) F-actin/DAPI staining of BMSCs-loaded nanofilm are showcased across different groups: control, PLGA-GT, PLGA@ICA-GT, PLGA-GT@KGN and PLGA@ICA-GT@KGN, after 1 and 5 days of *in vitro* culture. Quantitative analysis of cell viability and OD value is conducted using the CCK8 assay for various BMSCs-loaded nanofilms after 1 and 5 days of *in vitro* culture (*P < 0.05; NS, no statistical significance).

Thereafter, BMSCs were seeded onto these nanofilms and cultured *in vitro* for 5 days. Live/dead cell staining revealed robust viability and efficient proliferation of BMSCs across all nanofilm groups (PLGA-GT, PLGA@ICA-GT, PLGA-GT@KGN and PLGA@ICA-GT@KGN) compared to the control group during the initial 1–5 days post-seeding. Notably, there were minimal red-stained dead cells observed in all five groups (Figure 2A). F-actin/DAPI staining illustrated well-organized cytoskeletal structures of BMSCs within all scaffold groups, indicating favorable cell proliferation from Day 1 to Day 5 (Figure 2B). Quantitative analysis of cell viability showed close to 100% viability among all five groups (Figure 2C). Results from the CCK8 assay showcased an increasing optical density (OD) value from Day 1 to Day 5 across all nanofilms (Figure 2D), indicating an excellent proliferation rate of BMSCs seeded onto these nanofilms.

In addition, live/dead cell staining revealed robust viability and efficient proliferation of chondrocytes across all nanofilms compared to the control group during the initial 1–5 days post-seeding. Notably, minimal red-stained dead cells were observed in all five groups (Supplemental Figure S2A). Quantitative analysis of cell viability showed close to 100% viability among all five groups (Supplemental Figure S2B) and showcased an increasing OD value from Day 1 to Day 5 across all nanofilms (Supplemental Figure S2C). Collectively, these results demonstrate the gratifying biocompatibility of our synthesized nanofilms with both BMSCs and chondrocytes.

### Evaluation of *in vitro* anti-inflammatory properties

**Figure 3.**
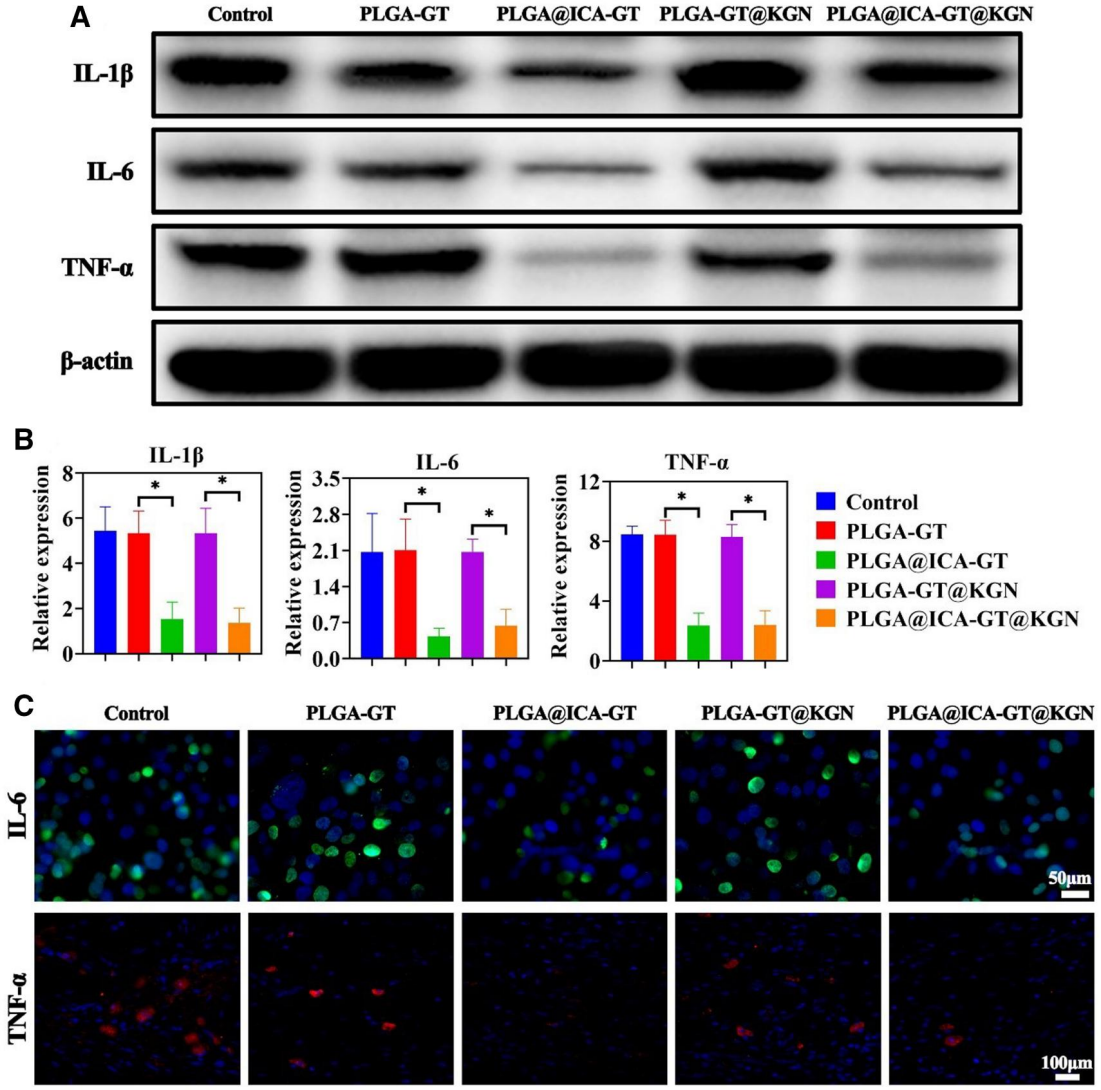
*In vitro* anti-inflammatory assessment of the PLGA@ICA-GT@KGN nanofilm on LPS-stimulated RAW264.7 macrophages. (**A**) Inflammatory-related protein expression (IL-1β, IL-6 and TNF-α) in different groups—control, PLGA-GT, PLGA@ICA-GT, PLGA-GT@KGN and PLGA@ICA-GT@KGN—is examined after 24 h of *in vitro* coculture via WB examination. (**B**) Inflammatory-related gene expression (*IL-1β*, *IL-6* and *TNF-α*) in various groups after 24 h of *in vitro* co-culture is analyzed using qRT-PCR (*P < 0.05). (**C**) Immunofluorescence staining for IL-6 and TNF-α in various groups after 24 h of *in vitro* co-culture.

LPS-induced RAW264.7 macrophages were co-cultured with the PLGA@ICA-GT@KGN nanofilm for 24 h. WB analysis was conducted to assess the expression levels of inflammatory-related proteins (IL-1β, IL-6 and TNF-α). The WB results depicted significantly lower expression levels of inflammatory-related proteins (IL-1β, IL-6 and TNF-α) in groups containing ICA (PLGA@ICA-GT and PLGA@ICA-GT@KGN nanofilms) compared to groups lacking ICA (control, PLGA-GT and PLGA-GT@KGN groups) (Figure 3A). qRT-PCR analysis complemented the WB results, demonstrating notably reduced expression levels of *IL-1β*, *IL-6* and *TNF-α* genes in ICA-containing groups (PLGA@ICA-GT and PLGA@ICA-GT@KGN nanofilms) in contrast to groups lacking ICA (control, PLGA-GT and PLGA-GT@KGN groups) (Figure 3B). In addition, immunofluorescence staining further confirmed the suppressed expression of IL-6 and TNF-α in ICA-containing groups compared to groups lacking ICA (Figure 3C).

### Evaluation of *in vitro* chondrogenic effects

**Figure 4.**
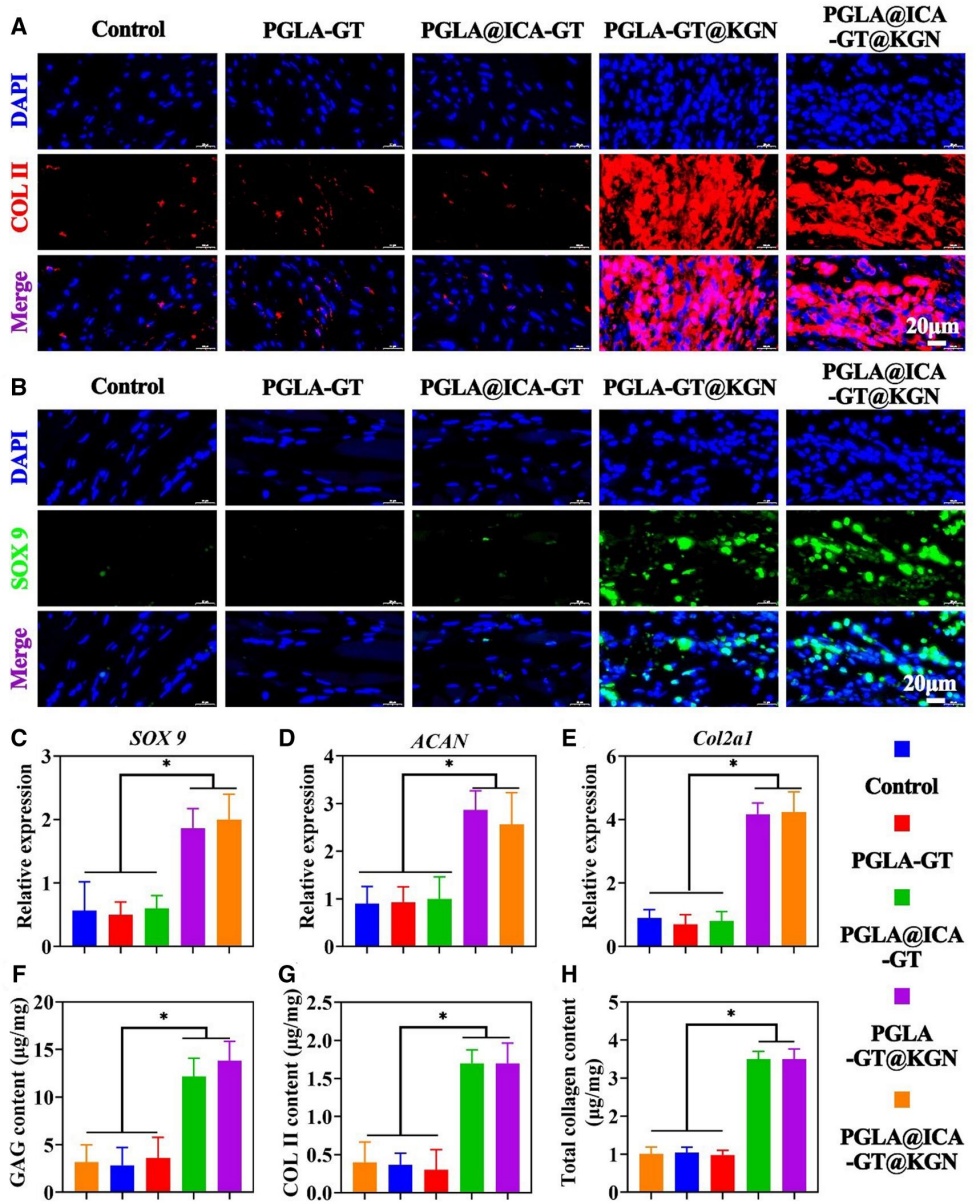
*In vitro* chondrogenic assessment of pretreated PLGA@ICA-GT@KGN nanofilm on BMSCs. (**A**) Immunofluorescence staining for COL II of BMSC-loaded nanofilms is depicted across different groups—control, PLGA-GT, PLGA@ICA-GT, PLGA-GT@KGN and PLGA@ICA-GT@KGN—after 14 days of *in vitro* culture. (**B**) Immunofluorescence staining for SOX9 of BMSC-loaded nanofilms is presented across the same groups after 14 days of *in vitro* culture. (**C**–**E**) Gene expression levels of chondrogenic-related markers (*SOX9*, *ACAN* and *Col2a1*) in various groups are examined after 14 days of *in vitro* culture. (**F**, **G**) Quantitative analysis of GAG, COL II and total collagen contents in different groups is conducted after 14 days of *in vitro* culture (*P < 0.05).

To assess the *in vitro* chondrogenic effects of the PLGA@ICA-GT@KGN nanofilm, the nanofilm was initially incubated in PBS for 14 days to promote the release of KGN, followed by coculturing with BMSCs for an additional 14 days. Immunofluorescence staining for COL II and SOX9 proteins revealed significantly higher expression levels in groups containing KGN (PLGA-GT@KGN and PLGA@ICA-GT@KGN nanofilms) compared to those without KGN (control, PLGA-GT and PLGA@ICA-GT groups) (Figure 4A and B). qRT-PCR analysis corroborated these findings, showing markedly increased expression levels of chondrogenic-related genes (*SOX9*, *ACAN* and *Col2a1*) in KGN-containing groups (PLGA-GT@KGN and PLGA@ICA-GT@KGN nanofilms) compared to groups lacking KGN (control, PLGA-GT and PLGA@ICA-GT groups) (Figure 4C–E). The expression levels of cartilage-specific proteins (GAG, COL II and total collagen) exhibited a consistent trend with the results from immunofluorescence staining and qRT-PCR analyses (Figure 4F–H).

### Evaluation of *in vivo* anti-inflammatory performance

**Figure 5.**
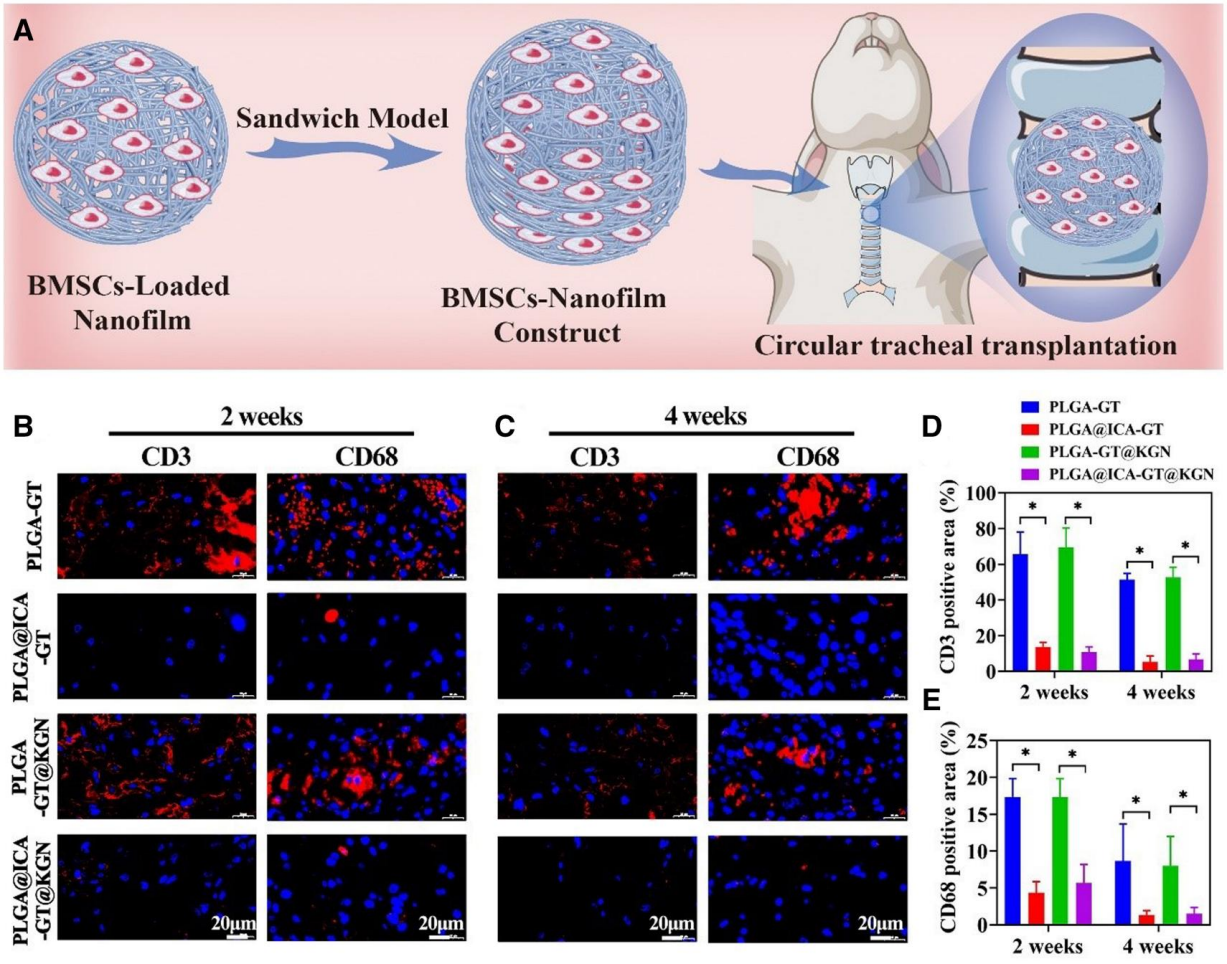
*In vivo* anti-inflammatory evaluation. (**A**) A schematic illustrates the preparation of the BMSCs-nanofilm construct using a ‘sandwich model’, which was subsequently employed for repairing circular tracheal defects. (**B**) Immunofluorescence staining for CD3 and CD68 is displayed in samples from the PLGA-GT, PLGA@ICA-GT, PLGA-GT@KGN and PLGA@ICA-GT@KGN groups after 2 weeks of tracheal transplantation. (**C**) Immunofluorescence staining for CD3 and CD68 in the same groups is shown after 4 weeks of tracheal transplantation. The percentages of (**D**) CD3 and (**E**) CD68 positive areas are quantified in the PLGA-GT, PLGA@ICA-GT, PLGA-GT@KGN and PLGA@ICA-GT@KGN groups at 2 and 4 weeks (*P < 0.05).

Following the successful demonstration of promising *in vitro* anti-inflammatory and chondrogenic properties of the PLGA@ICA-GT@KGN nanofilm, a BMSCs-loaded PLGA@ICA-GT@KGN nanofilm was constructed into a BMSCs-nanofilm composite using a ‘sandwich model’. This composite was utilized to repair circular tracheal defects (Figure 5A).

The *in vivo* anti-inflammatory effect of the PLGA@ICA-GT@KGN nanofilm was evaluated. Immunofluorescence staining for CD3 and CD68 revealed significantly lower expression levels of inflammation-related responses in groups containing ICA (PLGA@ICA-GT and PLGA@ICA-GT@KGN nanofilms) compared to groups lacking ICA (control, PLGA-GT and PLGA-GT@KGN groups) at both 2 and 4 weeks post-surgery (Figure 5A and B). Quantitative analysis of inflammation-positive areas further supported these findings, indicating statistically significant differences (Figure 5C and D). Notably, while the PLGA-GT and PLGA-GT@KGN groups exhibited a decreasing trend in CD3 and CD68 expression levels over time, these levels remained substantially higher than those observed in the PLGA@ICA-GT and PLGA@ICA-GT@KGN groups.

### Assessment of tracheal cartilage restoration *in vivo*

**Figure 6.**
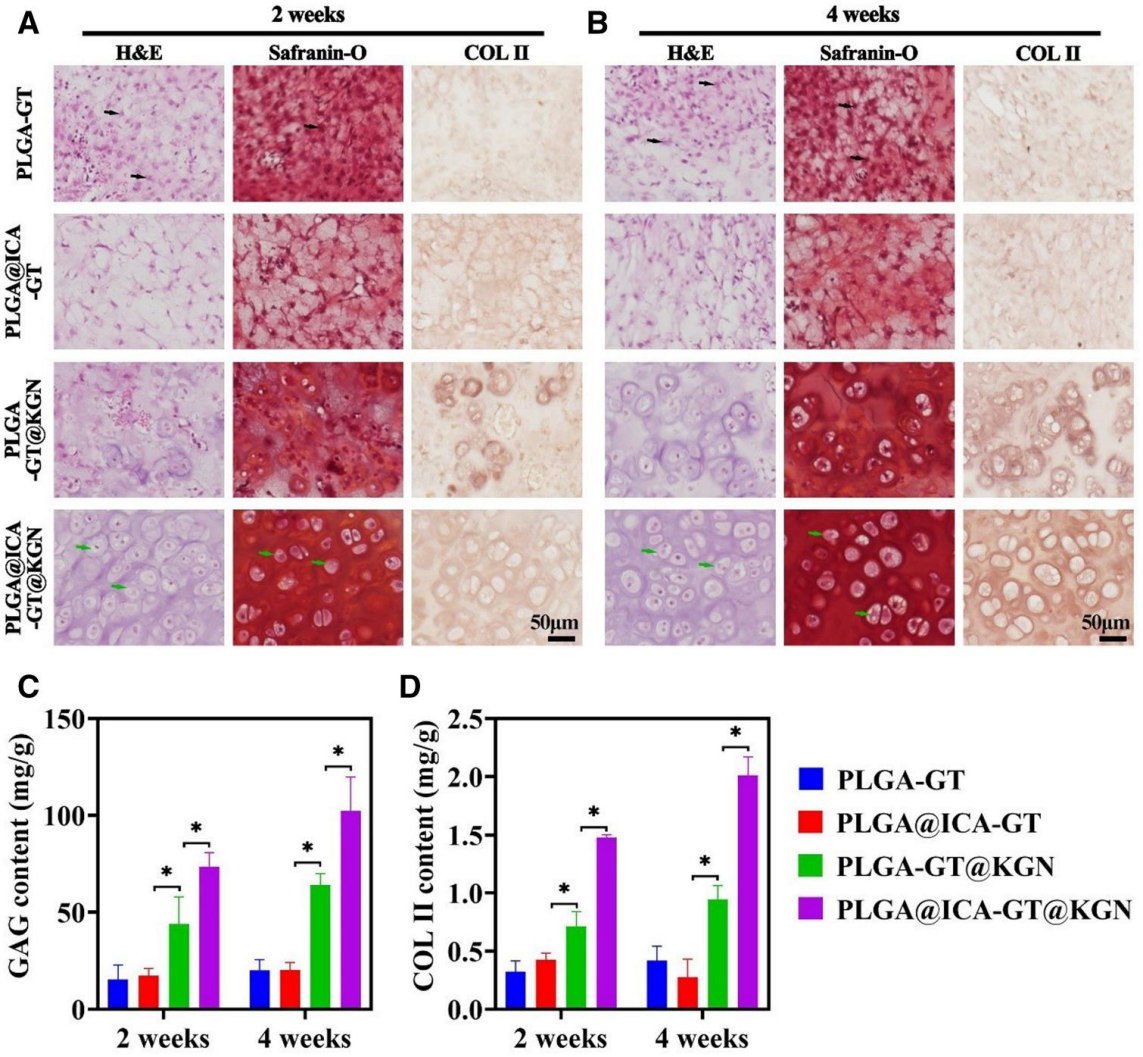
*In vivo* evaluation of cartilage regeneration. (**A**) H&E, Safranin-O and COL II staining in samples from the PLGA-GT, PLGA@ICA-GT, PLGA-GT@KGN and PLGA@ICA-GT@KGN groups after 2 weeks of tracheal transplantation. (**B**) H&E, Safranin-O and COL II staining in samples from the same groups after 4 weeks of tracheal transplantation. Inflammatory cells are indicated by black arrows, while chondrocytes are indicated by green arrows. Quantitative analysis of GAG and COL II content is conducted in the PLGA-GT, PLGA@ICA-GT, PLGA-GT@KGN and PLGA@ICA-GT@KGN groups at 2 and 4 weeks (*P < 0.05).

Following the established *in vivo* anti-inflammatory efficacy of the PLGA@ICA-GT@KGN nanofilm, the focus shifted to its potential in repairing tracheal cartilage defects. In the current study, the prepared sandwich construct was utilized to repair circular tracheal defects in a rabbit model (Figure 6A). Our results revealed a highest survival rate of experimental rabbits in PLGA@ICA-GT@KGN group (70%) post 8 weeks transplantation, whereas rabbits in PLGA@ICA-GT, PLGA-GT@KGN and PLGA-GT groups were all dead at 5, 4 and 3 weeks (Supplemental Figure S3A). Our data also suggested a superior healing tracheal condition of experimental rabbits in PLGA@ICA-GT@KGN group compared to PLGA@ICA-GT, PLGA-GT@KGN and PLGA-GT groups (Supplemental Table S1).

The tracheoscopy and photographs of the obtained repaired tracheal graft at 2 weeks showcased smooth lumen condition in PLGA@ICA-GT@KGN group, various degrees of granulated tissue in PLGA@ICA-GT, PLGA-GT@KGN and PLGA-GT groups (Supplemental Figure S3B,C). MicroCT examination revealed obvious extensive cartilage ECM deposition in PLGA@ICA-GT@KGN group, fewer cartilage ECM deposition PLGA@ICA-GT and PLGA-GT@KGN groups, whereas scarce cartilage ECM deposition in PLGA-GT groups (Supplemental Figure S4).

Histological assessments, including H&E, Safranin-O and COL II staining, revealed notable findings. The PLGA@ICA-GT@KGN group exhibited significantly higher counts of cartilage lacunae, specific ECM deposition and COL II deposition compared to the PLGA-GT, PLGA@ICA-GT and PLGA-GT@KGN groups at both 2 and 4 weeks (Supplemental Figure S5 and Figure 6B). Contrastingly, while the PLGA-GT and PLGA-GT@KGN groups displayed an increased presence of inflammation-related cells, the PLGA@ICA-GT group showed minimal inflammation-related cell generation but lacked significant cartilage regeneration. Quantitative measurements further confirmed the highest levels of glycosaminoglycan (GAG) and COL II contents in the PLGA@ICA-GT@KGN group compared to the other groups at both 2 and 4 weeks. Additionally, there was an observed increase in the PLGA@ICA-GT@KGN group over time, from 2 to 4 weeks (Figure 6C and D).

## Discussion

The healthy tracheal cartilage is essential for the proper function of the normal trachea, such as ventilation and expectoration. Consider the trachea as vital to life, thus, over the recent decades, researcher all over the world have been focused on finding the ideal tracheal cartilage substitutes. Numerous tracheal cartilage substitutes have been conceived, including autocostal cartilage, allogeneic trachea cartilage and even artificial cartilage protheses [32]. However, all these tracheal substitutes failed to achieve reliable therapeutic outcomes, mainly due to inferior tracheal cartilage restoration. Increasing evidences supported that tissue-engineered cartilage, which integrates cell, biomaterials and bioactive factors, is a promising avenue to develop a bioactive tracheal cartilage substitute with tailored biomechanical and biofunctions.

While tissue engineering for cartilage formation is well-established, a critical challenge lies in preserving the cartilage-specific ECM and functionality of transplanted tracheal substitutes in immunocompetent animals [33]. The inflammatory response stands as a primary obstacle to the therapeutic success of engineered cartilage in repairing tracheal defects [34]. One of the most direct evidence is the numerous articles reporting that robust cartilage regeneration can be easily achieved in nude mice without immune system; however, it is difficult to achieve the same good quality of cartilage regeneration in large animals with immunocompetent system [35, 36]. Studies indicate that cells cultured *in vitro* are susceptible to oxidative damage due to passaging and ectopic subcutaneous transplantation, leading to a partial loss of bioactivity in their surface membrane proteins [37]. Consequently, upon autologous transplantation, these cells can elicit an immune inflammatory response in the host [38]. The surgery to transplant tissue-engineered trachea is highly traumatic, also leading to inevitable inflammation. In addition, as heterogenic substances, degradation products of the materials within the tissue-engineered trachea can also cause inflammatory reactions. Moreover, the trachea itself is connected with the outside world, which is more likely to cause infection, and aggravate the inflammation of cartilage tissue corrosion. These inflammation responses pose detrimental effects on engineered tracheal cartilage substitutes and impede the tracheal repair process.

The inflammatory response typically involves the release of cytokines and activation of immune cells, which may lead to the following issues: (1) Cytokines released during inflammation can cause damage or cell death to chondrocytes, impeding the success of cartilage tissue engineering; (2) The presence of inflammatory mediators can also disrupt the normal function and growth of chondrocytes, disrupting or hindering the repair and regeneration of tracheal cartilage; (3) Inflammation may prompt excessive proliferation of fibroblasts, potentially resulting in scar tissue formation that affects the normal structure and function of repaired tracheal cartilage. Hence, controlling inflammation, reducing cellular damage caused by inflammation, and promoting cartilage repair processes are essential for optimizing the success of tracheal defect restoration. For instance, Yang *et al*. [34] prepared a porous curcumin-loaded polycaprolactone scaffold capable of anti-inflammation to enhance tracheal cartilage regeneration. Li *et al*. [39] developed a curcumin loaded hydrogel with anti-inflammatory activity to promote cartilage regeneration in immunocompetent animals. In this study, we proposed the encapsulation of ICA to endow the PLGA@ICA-GT@KGN nanofilm with potent anti-inflammatory. Both *in vitro* and *in vivo* results corroborate the PLGA@ICA-GT@KGN nanofilm could effectively release ICA to exert anti-inflammatory effect, laying the foundation for cartilage regeneration.

The sole anti-inflammatory niche cannot guarantee good cartilage regeneration based on BMSCs. It is well known that the cartilage regeneration using stem cells requires a favorable chondrogenic microenvironment. Hence, to promote the BMSCs-based tracheal cartilage formation, it is necessary to introduce a biological factor with good chondrogenic activity. Herein, KGN, known for its potent chondrogenic induction capability, was loaded into the PLGA@ICA-GT@KGN nanofilm. Our *in vitro* and *in vivo* results confirmed the PLGA@ICA-GT@KGN nanofilm could effectively release KGN to impart chondrogenic effect, giving rise to excellent tracheal cartilage regeneration.

This study also highlights a sequential regulatory of initially exhibiting anti-inflammatory properties and subsequently inducing chondrogenesis. The core principle is an immunosuppressive microenvironment is a prerequisite for cartilage regeneration. In the early stage of cartilage development, the immatured cartilage tissue featured with scarce ECM, the BMSCs and/or chondrocytes were directly exposed to immunocytes, which causing damage or cell death to these cells and disrupt the normal function and growth of these cells, thus severely hinder the chondrogenic efficacy [40, 41]. Hence, this study proposed to develop a shell–core structured PLGA@ICA-GT@KGN nanofilm. The rationale behind this design lies in the initial release of ICA from the shell layer, aiming to impart anti-inflammatory effects. Subsequent release of KGN from the core layer is intended to promote chondrogenesis of BMSCs, thereby enhancing cartilage regeneration for the restoration of the trachea. Our *in vitro* release kinetic results confirmed the initial release of ICA at 0–15 days and gradually releasing KGN among 11–29 days.

One exceptional technique for crafting shell–core structured nanofilms is coaxial electrospinning. This method manipulates two distinct solutions to create a nanofilm with a shell–core structure. Adjusting parameters like solution concentration, voltage, or spinneret-to-collector distance directly influences the thickness, porosity and morphology of the shell layer, thereby impacting the release rate and temporal control of growth factors. The resulting shell–core structure effectively shields growth factors from external influences, prolonging their efficacy within the body and facilitating controlled release. The temporal degradation effects of this structure enable the staged release of growth factors in specific shell and core layers [42, 43]. By modulating the degradation rate or adjusting the shell structure, growth factors can be continuously released over specific time frames. Ultimately, electrospun shell–core structures provide precise control over growth factor release. The ability to tailor and adjust the shell structure offers temporal regulation of growth factor release, presenting a robust tool for both medical treatments and biological research.

The incorporation of ICA demonstrated excellent anti-inflammatory effects following *in situ* tracheal restoration. This highlights the creation of an immunosuppressive microenvironment, a crucial prerequisite for tracheal cartilage regeneration. The potential release of ICA from the structured shell–core PLGA@ICA-GT@KGN nanofilm, contributing to its anti-inflammatory effect. ICA, a natural compound primarily found in the Chinese herb Epimedium, demonstrates notable anti-inflammatory properties [44]. Firstly, it effectively inhibits oxidative stress reactions, mitigating the generation and harm caused by free radicals [45]. Moreover, at certain concentrations, ICA has been observed to enhance the viability of TNF-α-induced inflammatory chondrocytes without causing toxicity to cartilage cells, as discovered by Mi *et al*. [46]. Additionally, ICA significantly diminishes the expression of pro-inflammatory cytokines (IL-1β, IL-6 and TNF-α) [47], inhibits the levels of nitric oxide and reactive oxygen species, thereby exerting an anti-inflammatory effect and restoring cartilage metabolic balance. ICA can also upregulate antioxidant signaling pathways like Nrf2/ARE/HO-1, which contribute to the clearance of free radicals and oxidative stress products in the body, thereby mitigating inflammation-induced tissue damage [48]. Studies also reveal ICA's capacity to alleviate LPS-induced inflammatory reactions through the NF-κB signaling pathway. In experiments where ICA was introduced into a culture medium containing TNF-α, it effectively suppressed the inflammatory response initiated by TNF-α by downregulating the NF-κB/HIF-2α signaling pathway in chondrocytes [49]. ICA can also suppress signaling pathways mediated by NLRP3/caspase-1/IL-1β, STAT and MAPK, which are crucial in regulating inflammatory responses. By inhibiting the activation of these pathways, ICA can impede the further development of inflammation [50]. Consequently, ICA emerges as a potent and effective anti-inflammatory agent.

Our findings also underscore the favorable chondrogenic induction effect of the PLGA@ICA-GT@KGN nanofilm. They further support the idea of KGN release from the core layer of the nanofilm, contributing to its chondrogenic induction capabilities. KGN, a small molecule, stimulates the chondrogenic differentiation of BMSCs. KGN upregulates the levels of COL II, aggrecan and MMP tissue inhibitors I, underscoring its role in preserving the phenotype of chondrocytes and safeguarding the cartilage ECM from degradation [51]. Research indicates that KGN promotes the chondrogenic differentiation of BMSCs by interacting with filamin A (FLNA), a cytoskeletal protein that links to actin. KGN binds to the FC-1 fragment of FLNA, causing the release of core-binding factor subunit beta (CBFβ) from its cytoplasmic binding site [52, 53]. Subsequently, CBFβ translocates into the nucleus where it associates with runt-related transcription factor (Runx) factors, thereby orchestrating the transcriptional program governing the expression of proteins and genes related to cartilage formation. Moreover, it is known that Runx2 can guide the differentiation of osteoblasts and hypertrophy of chondrocytes, thus contributing to the pathogenesis of osteoarthritis [54]. The CBFβ-Runx1 transcriptional program may also help maintain relatively low levels of Runx2, thereby preserving the functionality of cartilage formation [55]. These findings suggest that KGN may promote chondrogenesis of BMSCs through Runx1-mediated transcriptional effects, partly via activation of the TGF-β1-Smad2/3 pathway and inhibition of Runx2 expression [56, 57].

In this study, ICA and PLGA blend was used as shell layer, whereas KGN and GT blend was utilized as core layer, respectively. All these materials are well-established for their safety and compatibility. This combination was used to create the shell–core structured PLGA@ICA-GT@KGN nanofilm.

The selection of seed cells is of great significance for tracheal cartilage repair [58]. BMSCs, a commonly used cell type with definite chondrogenic capacity, were employed as seeding cells [59]. BMSCs can express various surface antigens, such as CD71, CD44, CD105, CD90 and SH2, SH3, etc. They do not express fibroblast labeled antigen Laminine, hematopoietic stem cell labeled antigen CD34, leukocyte labeled antigen CD45 and lymphocyte surface antigen CD11b [60, 61]. CD44 and CD90 were selected as identification markers in this experiment. The results of immunofluorescence staining confirmed that the cells we isolated and cultured only expressed CD44 and CD90, while the hematopoietic stem cell marker antigen CD34 showed negative expression, indicating that the cells we isolated and cultured were BMSCs with relatively single components, rather than hematopoietic stem cells and fibroblasts. Immunofluorescence staining also showed that the BMSCs we isolated and cultured were highly expressed in CD90, which indicates that the BMSCs are in a primitive undifferentiated state [62]. The antigen indicates that the isolated cells are of a very young biological age and have distinct stem cell characteristics.

Biocompatibility stands as a fundamental trait in a tissue engineering scaffold. The results obtained from live/dead cell staining and CCK-8 quantification demonstrate favorable biocompatibility of PLGA@ICA-GT@KGN nanofilm to both BMSCs and chondrocytes. F-actin/DAPI staining further confirms satisfactory BMSCs diffusion in the PLGA@ICA-GT@KGN nanofilm. The safety and non-toxic nature of PLGA and GT, validated extensively for their biocompatibility, along with the safety profiles of ICA and KGN, indirectly affirm the reliable and safe dosages of drugs used in this study. Collectively, these results demonstrated the gratifying biocompatibility of our synthesized PLGA@ICA-GT@KGN nanofilms to both BMSCs and chondrocytes, highlighting their potential for use in tracheal cartilage regeneration.

The ultimate aim of this study lies in evaluating the *in vivo* anti-inflammatory and tracheal cartilage repair effects. Considering the single layer of BMSCs-loaded nanofilm were ultrathin, failing to match the cartilage thickness and mechanical strength requirements of normal tracheal cartilage. Previous studies have demonstrated that stacking BMSCs and nanofilms using a ‘sandwich model’ helps achieve closer contact between BMSCs and nanofilms, enabling drugs and factors to fully exert their functions [63]. Hence, this study proposes to form a BMSCs-nanofilm construct via the four-layer superposition of BMSCs-loaded nanofilm based on ‘sandwich model’, aiming to increase the thickness of the resultant BMSCs-nanofilm construct. Examination following implantation of the BMSCs-nanofilm construct into a rabbit tracheal defect model revealed intriguing outcomes. Compared to the PLGA-GT group, the PLGA@ICA-GT group (ICA alone added) achieved remarkable *in vivo* anti-inflammatory effects but showed minimal cartilage fossa formation and lacked specific cartilage ECM deposition. This underscores that establishing an anti-inflammatory microenvironment alone *in vivo* is insufficient for repairing tracheal cartilage defects. Conversely, the PLGA-GT@KGN group (only KGN added) displayed minimal *in vivo* cartilage regeneration, accompanied by increased inflammation-related cells and significantly higher inflammatory factor expression compared to the ICA-added group. In contrast, the PLGA@ICA-GT@KGN group (both ICA and KGN added) exhibited no expression of inflammatory factors, robust cartilage fossa regeneration and specific cartilage ECM deposition. These outcomes validate our hypothesis that a sequential regulatory strategy, beginning with an anti-inflammatory microenvironment and followed by chondrogenic induction, achieves satisfactory cartilage regeneration *in vivo*, facilitating *in situ* repair of tracheal cartilage defects.

## Conclusion

This study developed a structured PLGA@ICA-GT@KGN nanofilm with sequential regulatory capabilities. This nanofilm, comprising ICA and KGN bioactive compounds, enables controlled and sequential release—initially, releasing the anti-inflammatory agent ICA followed by the cartilage-inducing substance KGN. *In vitro* experiments demonstrated the significant anti-inflammatory effect of ICA release and the sustained KGN release's promotion of BMSCs' chondrogenic induction. In addition, the BMSC-loaded nanofilms were assembled into a sandwich structure, and was then used to repair circular tracheal defect. The therapeutic outcomes confirmed the nanofilm's sequential regulatory function, showcasing remarkable anti-inflammatory properties and successful tracheal cartilage restoration. Consequently, the PLGA@ICA-GT@KGN nanofilm scaffold, adept at sequentially mediating anti-inflammatory and cartilage-inducing effects, emerges as a promising solution for repairing tracheal cartilage defects. Its potential addresses key challenges in clinical tracheal cartilage injuries, signifying broad application prospects.

## Supplementary Material

rbae040_Supplementary_Data

## References

[1] Lee HY , Lee JW. Current status and future outlook of additive manufacturing technologies for the reconstruction of the trachea. J Funct Biomater 2023;14(4):196. 10.3390/jfb14040196 PMC10141199 37103286

[2] Soriano L , Khalid T, Whelan D, O'Huallachain N, Redmond KC, O'Brien FJ, O'Leary C, Cryan SA. Development and clinical translation of tubular constructs for tracheal tissue engineering: a review. Eur Respir Rev 2021;30(162):210154. 10.1183/16000617.0154-2021 PMC9488721 34750116

[3] Rostamani H , Fakhraei O, Zamirinadaf N, Mahjour M. An overview of nasal cartilage bioprinting: from bench to bedside. J Biomater Sci Polym Ed 2024.2321636. 10.1080/09205063.2024.2321636 38441976

[4] Shuai C , Yang W, Feng P, Peng S, Pan H. Accelerated degradation of HAP/PLLA bone scaffold by PGA blending facilitates bioactivity and osteoconductivity. Bioact Mater 2021;6:490–502. 32995675 10.1016/j.bioactmat.2020.09.001 PMC7493133

[5] Feng P , Wu P, Gao C, Yang Y, Guo W, Yang W, Shuai C. A multimaterial scaffold with tunable properties: toward bone tissue repair. Adv Sci (Weinh) 2018;5:1700817. 29984132 10.1002/advs.201700817 PMC6033191

[6] Han J , Rindone AN, Elisseeff JH. Immunoengineering biomaterials for musculoskeletal tissue repair across lifespan. Adv Mater 2024;e2311646. 38416061 10.1002/adma.202311646 PMC11239302

[7] Cardoneanu A , Rezus II, Burlui AM, Richter P, Bratoiu I, Mihai IR, Macovei LA, Rezus E. Autoimmunity and autoinflammation: relapsing polychondritis and VEXAS syndrome challenge. Int J Mol Sci 2024;25(4):2261. 10.3390/ijms25042261 PMC10889424 38396936

[8] Khoshneviszadeh M , Henneicke S, Pirici D, Senthilnathan A, Morton L, Arndt P, Kaushik R, Norman O, Jukkola J, Dunay IR, Seidenbecher C, Heikkinen A, Schreiber S, Dityatev A. Microvascular damage, neuroinflammation and extracellular matrix remodeling in Col18a1 knockout mice as a model for early cerebral small vessel disease. Matrix Biol 2024(128):39–64. 38387749 10.1016/j.matbio.2024.02.007

[9] Byun H , Han Y, Kim E, Jun I, Lee J, Jeong H, Huh SJ, Joo J, Shin SR, Shin H. Cell-homing and immunomodulatory composite hydrogels for effective wound healing with neovascularization. Bioact Mater 2024;36:185–202. 38463552 10.1016/j.bioactmat.2024.02.029 PMC10924181

[10] Shuai CJ , Guo W, Wu P, Yang WJ, Hu S, Xia Y, Feng P. A graphene oxide-Ag co-dispersing nanosystem: dual synergistic effects on antibacterial activities and mechanical properties of polymer scaffolds. Chem Eng J 2018;347:322–33.

[11] Fan R , Zhao J, Yi L, Yuan J, McCarthy A, Li B, Yang G, John JV, Wan W, Zhang Y, Chen S. Anti-inflammatory peptide-conjugated silk fibroin/cryogel hybrid dual fiber scaffold with hierarchical structure promotes healing of chronic wounds. Adv Mater 2024;36:2307328. 10.1002/adma.202307328 38288789

[12] Luo Y , Xiao M, Almaqrami BS, Kang H, Shao Z, Chen X, Zhang Y. Regenerated silk fibroin based on small aperture scaffolds and marginal sealing hydrogel for osteochondral defect repair. Biomater Res 2023;27:50. 37208690 10.1186/s40824-023-00370-1 PMC10197849

[13] Celikkin N , Presutti D, Maiullari F, Volpi M, Promovych Y, Gizynski K, Dolinska J, Wiśniewska A, Opałło M, Paradiso A, Rinoldi C, Fuoco C, Swieszkowski W, Bearzi C, Rizzi R, Gargioli C, Costantini M. Combining rotary wet-spinning biofabrication and electro-mechanical stimulation for thein vitroproduction of functional myo-substitutes. Biofabrication 2023;15:045012. 10.1088/1758-5090/ace934 37473749

[14] Peredo AP , Gullbrand SE, Friday CS, Orozco BS, Dehghani B, Jenk AC, Bonnevie ED, Hilliard RL, Zlotnick HM, Dodge GR, Lee D, Engiles JB, Hast MW, Schaer TP, Smith HE, Mauck RL. Tension-activated nanofiber patches delivering an anti-inflammatory drug improve repair in a goat intervertebral disc herniation model. Sci Transl Med 2023;15:eadf1690. 37967202 10.1126/scitranslmed.adf1690 PMC10812087

[15] Li X , Sun S, Yang A, Li X, Jiang Z, Wu S, Zhou F. Dual-crosslinked methacrylamide chitosan/poly(ε-caprolactone) nanofibers sequential releasing of tannic acid and curcumin drugs for accelerating wound healing. Int J Biol Macromol 2023;253:127601. 37871718 10.1016/j.ijbiomac.2023.127601

[16] Peng L , Lu Z, Zhong L, Jian J, Rong Y, Yang R, Xu Y, Jin C. Enhanced ionic conductivity and interface compatibility of PVDF-LLZTO composite solid electrolytes by interfacial maleic acid modification. J Colloid Interface Sci 2022;613:368–75. 35042034 10.1016/j.jcis.2022.01.031

[17] Lee CH , Chen DY, Hsieh MJ, Hung KC, Huang SC, Cho CJ, Liu SJ. Nanofibrous insulin/vildagliptin core–shell PLGA scaffold promotes diabetic wound healing. Front Bioeng Biotechnol 2023;11:1075720. 37168611 10.3389/fbioe.2023.1075720 PMC10164987

[18] He W , Li C, Zhao S, Li Z, Wu J, Li J, Zhou H, Yang Y, Xu Y, Xia H. Integrating coaxial electrospinning and 3D printing technologies for the development of biphasic porous scaffolds enabling spatiotemporal control in tumor ablation and osteochondral regeneration. Bioact Mater 2024;34:338–53. 38274295 10.1016/j.bioactmat.2023.12.020 PMC10809007

[19] Dong Y , Wang L, Yang M, Zhou X, Li G, Xu K, Ma Y, Chen J, Wang Z, Zhou J, Li H, Zhu Z. Effect of icariin on depressive behaviour in rat pups. Evidences for its mechanism of action by integrating network pharmacology, metabolomics and gut microbiota composition. Phytomedicine 2024;126:155422. 38422651 10.1016/j.phymed.2024.155422

[20] Song LJ , Han QX, Ding ZB, Liu K, Zhang XX, Guo MF, Ma D, Wang Q, Xiao BG, Ma CG. Icariin ameliorates the cuprizone-induced demyelination associated with antioxidation and anti-inflammation. Inflammopharmacology 2024;32:809–23. 38177566 10.1007/s10787-023-01388-6

[21] Sonam Dongsar T , Tsering Dongsar T, Gupta G, Alsayari A, Wahab S, Kesharwani P. PLGA nanomedical consignation: a novel approach for the management of prostate cancer. Int J Pharm 2024;652:123808. 38224758 10.1016/j.ijpharm.2024.123808

[22] Zhang M , Hu W, Cai C, Wu Y, Li J, Dong S. Advanced application of stimuli-responsive drug delivery system for inflammatory arthritis treatment. Mater Today Bio 2022;14:100223. 10.1016/j.mtbio.2022.100223 PMC8881671 35243298

[23] Zhou J. Curcumin-loaded porous scaffold: an anti-angiogenic approach to inhibit endochondral ossification. J Biomater Sci Polym Ed 2023;34:2255–73. 37382577 10.1080/09205063.2023.2231663

[24] Huang Z , Zhang Y, Liu R, Li Y, Rafique M, Midgley AC, Wan Y, Yan H, Si J, Wang T, Chen C, Wang P, Shafiq M, Li J, Zhao L, Kong D, Wang K. Cobalt loaded electrospun poly(epsilon-caprolactone) grafts promote antibacterial activity and vascular regeneration in a diabetic rat model. Biomaterials 2022;291:121901. 36356473 10.1016/j.biomaterials.2022.121901

[25] Rafique M , Wei T, Sun Q, Midgley AC, Huang Z, Wang T, Shafiq M, Zhi D, Si J, Yan H, Kong D, Wang K. The effect of hypoxia-mimicking responses on improving the regeneration of artificial vascular grafts. Biomaterials 2021;271:120746. 33725586 10.1016/j.biomaterials.2021.120746

[26] Zhang HM , Guo M, Zhu TH, Xiong H, Zhu LM. A careob-like nanofibers with a sustained drug release profile for promoting skin wound repair and inhibiting hypertrophic scar. Compos Part B Eng 2022;236:109790.

[27] Yan H , Cheng Q, Si J, Wang S, Wan Y, Kong X, Wang T, Zheng W, Rafique M, Li X, He J, Midgley AC, Zhu Y, Wang K, Kong D. Functionalization of in vivo tissue-engineered living biotubes enhance patency and endothelization without the requirement of systemic anticoagulant administration. Bioact Mater 2023;26:292–305. 36950151 10.1016/j.bioactmat.2023.03.003 PMC10027480

[28] Zhang Y , Xu K, Zhi D, Qian M, Liu K, Shuai Q, Qin Z, Xie J, Wang K, Yang J. Improving vascular regeneration performance of electrospun poly(ε-caprolactone) vascular grafts via synergistic functionalization with VE-cadherin/VEGF. Adv Fiber Mater 2022;4:1685–702.

[29] Zhi D , Cheng Q, Midgley AC, Zhang Q, Wei T, Li Y, Wang T, Ma T, Rafique M, Xia S, Cao Y, Li Y, Li J, Che Y, Zhu M, Wang K, Kong D. Mechanically reinforced biotubes for arterial replacement and arteriovenous grafting inspired by architectural engineering. Sci Adv 2022;8:eabl3888. 35294246 10.1126/sciadv.abl3888 PMC8926343

[30] Xu Y , Dai J, Zhu X, Cao R, Song N, Liu M, Liu X, Zhu J, Pan F, Qin L, Jiang G, Wang H, Yang Y. Biomimetic trachea engineering via a modular ring strategy based on bone-marrow stem cells and atelocollagen for use in extensive tracheal reconstruction. Adv Mater 2022;34:e2106755. 34741771 10.1002/adma.202106755

[31] Xu Y , Duan H, Li YQ, She YL, Zhu JJ, Zhou GD, Jiang GN, Yang Y. Nanofibrillar decellularized wharton's jelly matrix for segmental tracheal repair. Adv Funct Materials 2020;30:1910067.

[32] Tanh NTV , Thach PN, Phong LS, Ngoc LTD, Nguyen PT, Van Anh Dung H, Mau TA, Nhan VT. Tracheal reconstruction surgery for congenital tracheal stenosis. Pediatr Surg Int 2023;39:123. 36787049 10.1007/s00383-023-05418-w

[33] Xu M , Su T, Jin X, Li Y, Yao Y, Liu K, Chen K, Lu F, He Y. Inflammation-mediated matrix remodeling of extracellular matrix-mimicking biomaterials in tissue engineering and regenerative medicine. Acta Biomater 2022;151:106–17. 35970482 10.1016/j.actbio.2022.08.015

[34] Yang ML , Sun WY, Wang L, Tang H, Xu X, Yang LW, Ni JJ, Zheng KE, Jiang X, Xu WW, Zhao GF, She YL, Zhang L, Xie D, Chen C. Curcumin loaded polycaprolactone scaffold capable of anti-inflammation to enhance tracheal cartilage regeneration. Mater Des 2022;224:111299.

[35] Xu Y , Li D, Yin Z, He A, Lin M, Jiang G, Song X, Hu X, Liu Y, Wang J, Wang X, Duan L, Zhou G. Tissue-engineered trachea regeneration using decellularized trachea matrix treated with laser micropore technique. Acta Biomater 2017;58:113–21. 28546133 10.1016/j.actbio.2017.05.010

[36] Xu Y , Wang Z, Hua Y, Zhu X, Wang Y, Duan L, Zhu L, Jiang G, Xia H, She Y, Zhou G. Photocrosslinked natural hydrogel composed of hyaluronic acid and gelatin enhances cartilage regeneration of decellularized trachea matrix. Mater Sci Eng C Mater Biol Appl 2021;120:111628. 33545814 10.1016/j.msec.2020.111628

[37] Fu JY , Jing Y, Xiao YP, Wang XH, Guo YW, Zhu YJ. Astaxanthin inhibiting oxidative stress damage of placental trophoblast cells in vitro. Syst Biol Reprod Med 2021;67:79–88. 33103484 10.1080/19396368.2020.1824031

[38] Tang SW , Tong WY, Pang SW, Voelcker NH, Lam YW. Deconstructing, replicating, and engineering tissue microenvironment for stem cell differentiation. Tissue Eng Part B Rev 2020;26:540–54. 32242476 10.1089/ten.TEB.2020.0044

[39] Li X , He L, Li N, He D. Curcumin loaded hydrogel with anti-inflammatory activity to promote cartilage regeneration in immunocompetent animals. J Biomater Sci Polym Ed 2023;34:200–16. 35971659 10.1080/09205063.2022.2113290

[40] Cho S , Discher DE, Leong KW, Vunjak-Novakovic G, Wu JC. Challenges and opportunities for the next generation of cardiovascular tissue engineering. Nat Methods 2022;19:1064–71. 36064773 10.1038/s41592-022-01591-3 PMC12061062

[41] Raghav PK , Mann Z, Ahlawat S, Mohanty S. Mesenchymal stem cell-based nanoparticles and scaffolds in regenerative medicine. Eur J Pharmacol 2022;918:174657. 34871557 10.1016/j.ejphar.2021.174657

[42] Liu L , Yu W, Seitsonen J, Xu W, Lehto VP. Correct identification of the core-shell structure of cell membrane-coated polymeric nanoparticles. Chemistry 2022;28:e202200947. 36116117 10.1002/chem.202200947 PMC10091812

[43] Sharda D , Choudhury D. Insulin-cobalt core–shell nanoparticles for receptor-targeted bioimaging and diabetic wound healing. RSC Adv 2023;13:20321–35. 37425626 10.1039/d3ra01473h PMC10323873

[44] He C , Wang Z, Shi J. Pharmacological effects of icariin. Adv Pharmacol 2020;87:179–203. 32089233 10.1016/bs.apha.2019.10.004

[45] Liu J , Cheng Q, Wu X, Zhu H, Deng X, Wang M, Yang S, Xu J, Chen Q, Li M, Liu X, Wang C. Icariin treatment rescues diabetes induced bone loss via scavenging ROS and activating primary cilia/Gli2/osteocalcin signaling pathway. Cells 2022;11:11. 10.3390/cells11244091 PMC9777100 36552853

[46] Mi B , Wang J, Liu Y, Liu J, Hu L, Panayi AC, Liu G, Zhou W. Icariin activates autophagy via down-regulation of the NF-κB signaling-mediated apoptosis in chondrocytes. Front Pharmacol 2018;9:605. 29950992 10.3389/fphar.2018.00605 PMC6008570

[47] Liu X , Wang Z, Qian H, Tao W, Zhang Y, Hu C, Mao W, Guo Q. Natural medicines of targeted rheumatoid arthritis and its action mechanism. Front Immunol 2022;13:945129. 35979373 10.3389/fimmu.2022.945129 PMC9376257

[48] Lu CS , Wu CY, Wang YH, Hu QQ, Sun RY, Pan MJ, Lu XY, Zhu T, Luo S, Yang HJ, Wang D, Wang HW. The protective effects of icariin against testicular dysfunction in type 1 diabetic mice via AMPK-mediated Nrf2 activation and NF-κB p65 inhibition. Phytomedicine 2024;123:155217. 37992492 10.1016/j.phymed.2023.155217

[49] Wang P , Meng Q, Wang W, Zhang S, Xiong X, Qin S, Zhang J, Li A, Liu Z. Icariin inhibits the inflammation through down-regulating NF-κB/HIF-2α signal pathways in chondrocytes. Biosci Rep 2020;40:BSR20203107. 10.1042/BSR20203107 PMC7685011 33155655

[50] Ding X , Zhao H, Qiao C. Icariin protects podocytes from NLRP3 activation by Sesn2-induced mitophagy through the Keap1-Nrf2/HO-1 axis in diabetic nephropathy. Phytomedicine 2022;99:154005. 35247669 10.1016/j.phymed.2022.154005

[51] Ren E , Chen H, Qin Z, Guan S, Jiang L, Pang X, He Y, Zhang Y, Gao X, Chu C, Zheng L, Liu G. Harnessing bifunctional ferritin with kartogenin loading for mesenchymal stem cell capture and enhancing chondrogenesis in cartilage regeneration. Adv Healthc Mater 2022;11:e2101715. 34997700 10.1002/adhm.202101715

[52] Yoshida N , Ogata T, Tanabe K, Li S, Nakazato M, Kohu K, Takafuta T, Shapiro S, Ohta Y, Satake M, Watanabe T. Filamin A-bound PEBP2beta/CBFbeta is retained in the cytoplasm and prevented from functioning as a partner of the Runx1 transcription factor. Mol Cell Biol 2005;25:1003–12. 15657428 10.1128/MCB.25.3.1003-1012.2005 PMC543995

[53] Hayek A , Kerstetter-Fogle AE, Sachlos E, Bollenbach T. Kartogenin: a game-changer in regenerative medicine. Regen Med 2012;7:475. 22993885

[54] O'Connor WJ , Botti T, Khan SN, Lane JM. The use of growth factors in cartilage repair. Orthoped Clin N Am 2000;31:399–409. 10.1016/s0030-5898(05)70159-0 10882466

[55] Music E , Klein TJ, Lott WB, Doran MR. Transforming growth factor-beta stimulates human bone marrow-derived mesenchymal stem/stromal cell chondrogenesis more so than kartogenin. Sci Rep 2020;10:8340. 32433527 10.1038/s41598-020-65283-8 PMC7239921

[56] Nishida T , Kubota S, Aoyama E, Takigawa M. Impaired glycolytic metabolism causes chondrocyte hypertrophy-like changes via promotion of phospho-Smad1/5/8 translocation into nucleus. Osteoarthritis Cartilage 2013;21:700–9. 23384547 10.1016/j.joca.2013.01.013

[57] Hou M , Zhang Y, Zhou X, Liu T, Yang H, Chen X, He F, Zhu X. Kartogenin prevents cartilage degradation and alleviates osteoarthritis progression in mice via the miR-146a/NRF2 axis. Cell Death Dis 2021;12:483. 33986262 10.1038/s41419-021-03765-x PMC8119954

[58] Yi M , Tang X, Liang S, He R, Huang T, Lin Q, Zhang R. Effect of microwave alone and microwave-assisted modification on the physicochemical properties of starch and its application in food. Food Chem 2024;446:138841. 38428082 10.1016/j.foodchem.2024.138841

[59] Lee E , Epanomeritakis IE, Lu V, Khan W. Bone marrow-derived mesenchymal stem cell implants for the treatment of focal chondral defects of the knee in animal models: a systematic review and meta-analysis. Int J Mol Sci 2023;24:3227. 36834639 10.3390/ijms24043227 PMC9958893

[60] Short B , Brouard N, Occhiodoro-Scott T, Ramakrishnan A, Simmons PJ. Mesenchymal stem cells. Arch Med Res 2003;34:565–71. 14734097 10.1016/j.arcmed.2003.09.007

[61] Pittenger MF , Mackay AM, Beck SC, Jaiswal RK, Douglas R, Mosca JD, Moorman MA, Simonetti DW, Craig S, Marshak DR. Multilineage potential of adult human mesenchymal stem cells. Science 1999;284:143–7. 10102814 10.1126/science.284.5411.143

[62] Ghilzon R , McCulloch CA, Zohar R. Stromal mesenchymal progenitor cells. Leuk Lymphoma 1999;32:211–21. 10037019 10.3109/10428199909167382

[63] Ojha AK , Rajasekaran R, Hansda AK, Singh A, Dutta A, Seesala VS, Das S, Dogra N, Sharma S, Goswami R, Chaudhury K, Dhara S. Biodegradable multi-layered silk fibroin-PCL stent for the management of cervical atresia: in vitro cytocompatibility and extracellular matrix remodeling in vivo. ACS Appl Mater Interfaces 2023;15:39099–116. 37579196 10.1021/acsami.3c06585

