# Supplementary material for: Temporal control in shell–core structured nanofilm for tracheal cartilage regeneration: synergistic optimization of anti-inflammation and chondrogenesis
Source: Regen Biomater. 2024 Apr 11;11:rbae040. doi: 10.1093/rb/rbae040 (PMC11105955; doi:10.1093/rb/rbae040)
Supplement: rbae040_Supplementary_Data [file rbae040_supplementary_data.docx]

**Supplemental Information**

**Temporal Control in Shell-core Structured Nanofilm for Tracheal Cartilage Regeneration: Synergistic Optimization of Anti-Inflammation and Chondrogenesis**

*Wen Zhao^1, 2#^, Fanglan Xu^1#^, Yumei Shen^3#^, Qifeng Ding^1^, Yifei Wang^1^, Leilei Liang^5*^, Wufei Dai^4*^, Yongbing Chen^1*^*

^1^ Department of Thoracic Surgery, the Second Affiliated Hospital of Soochow University, Suzhou, China

^2^ Department of Thoracic Surgery, Tongren Hospital, Shanghai Jiao Tong University, School of Medicine, Shanghai, China

^3^ Operation Room Department, the Second Affiliated Hospital of Soochow University, Suzhou, China

^4^ Department of Plastic and Reconstructive Surgery, Shanghai Ninth People’s Hospital, Shanghai Jiao Tong University School of Medicine, Shanghai, China

^5^ Department of Gynecologic Oncology, Zhejiang Cancer Hospital, Hangzhou, China.

^#^ These authors equally contributed to the work.

^*^ These authors contributed equally as corresponding authors.

Correspondence to: Dr. Leilei Liang, email: [liangleilei10006@163.com](mailto:liangleilei10006@163.com); Dr. Wufei Dai, email: [daiwfup@163.com](mailto:daiwfup@163.com); Prof. Yongbing Chen, email: chentongt@sina.com.


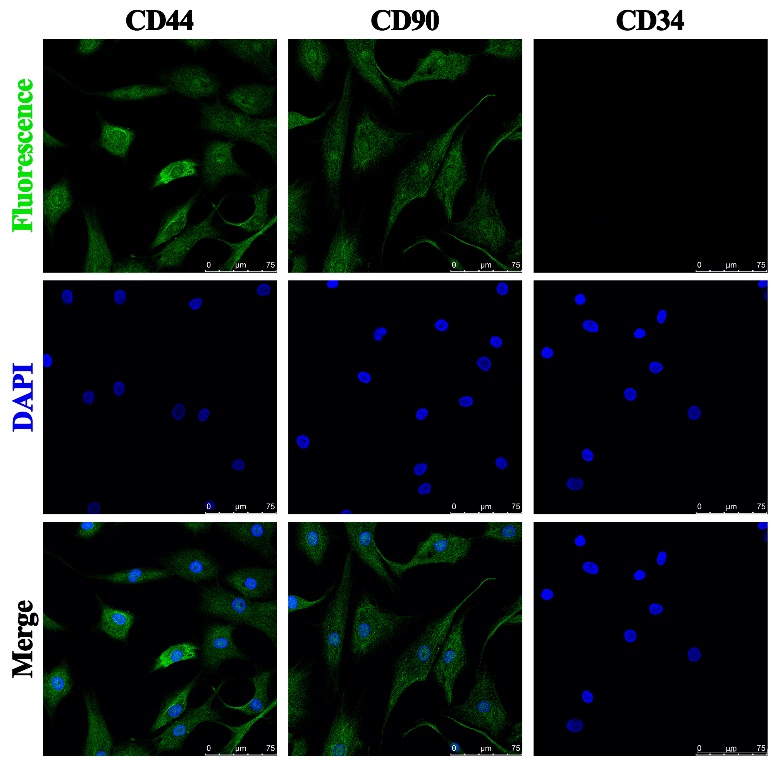


**Supplemental Figure 1.** Immunofluorescence staining for the expression of CD90, CD44, and CD34 in BMSCs.


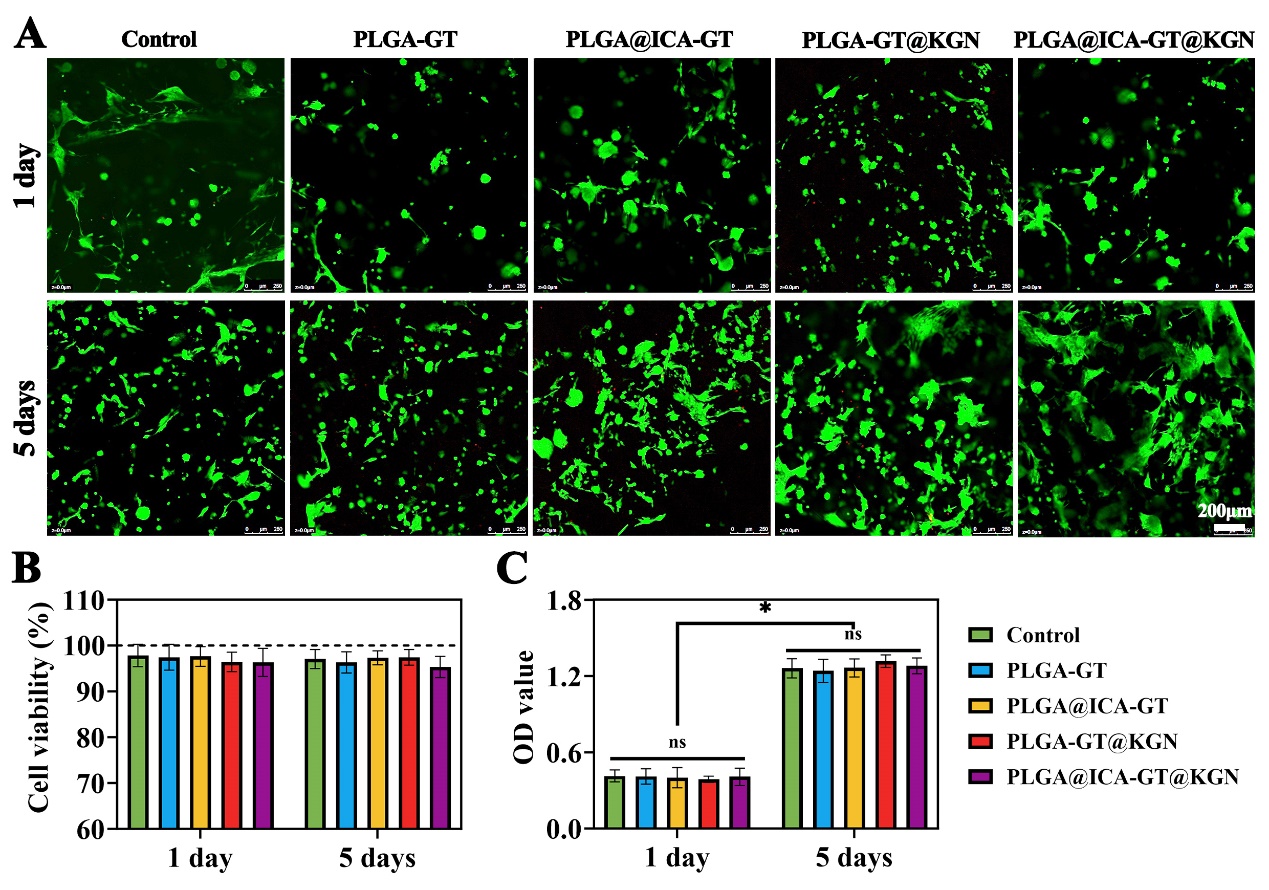


**Supplemental Figure 2.** **In vitro biocompatibility assessment of nanofilm on chondrocytes.** A) Live/dead staining of chondrocytes-loaded nanofilm are showcased across different groups: control, PLGA-GT, PLGA@ICA-GT, PLGA-GT@KGN, and PLGA@ICA-GT@KGN, after 1 and 5 days of in vitro culture. Quantitative analysis of cell viability and OD value is conducted using the CCK8 assay for various chondrocytes -loaded nanofilms after 1 and 5 days of in vitro culture (*P < 0.05; ns, no statistical significance).


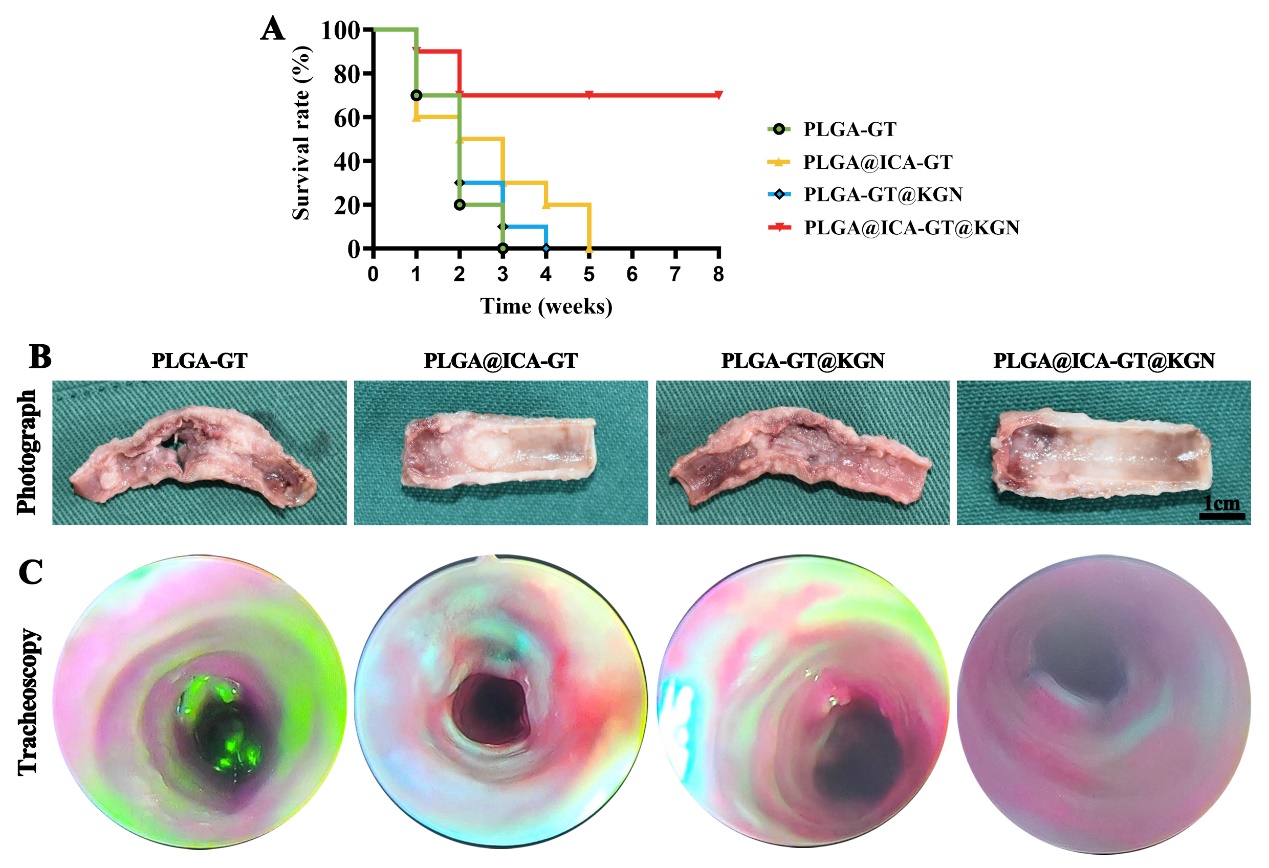


**Supplemental Figure 3.** Therapeutic outcome of BMSCs-nanofilm construct in treating circular tracheal defects. A) Survival rate of experimental rabbit in PLGA-GT, PLGA@ICA-GT, PLGA-GT@KGN, and PLGA@ICA-GT@KGN groups over 8 weeks. B) The represent photographs for the repaired trachea after 2 weeks. C) Tracheoscopy observation for repaired grafts in PLGA-GT, PLGA@ICA-GT, PLGA-GT@KGN, and PLGA@ICA-GT@KGN groups at 2 weeks.

**Supplemental Table1. The healing conditions**

| Application | BMSCs collection | In vivo repair | | | | | | | |
| --- | --- | --- | --- | --- | --- | --- | --- | --- | --- |
| Group | None | PLGA-GT | | PLGA@ICA-GT | | PLGA-GT@KGN | | PLGA@ICA-GT@KGN | |
|  |  | 2W | 4W | 2W | 4W | 2W | 4W | 2W | 4W |
| Healing Conditions  (%) | 100 | 20 | 22 | 22 | 24 | 41 | 56 | 70 | 98 |
|  | 100 | 18 | 25 | 20 | 25 | 38 | 63 | 75 | 94 |
|  |  | 15 | 19 | 23 | 20 | 42 | 70 | 74 | 89 |


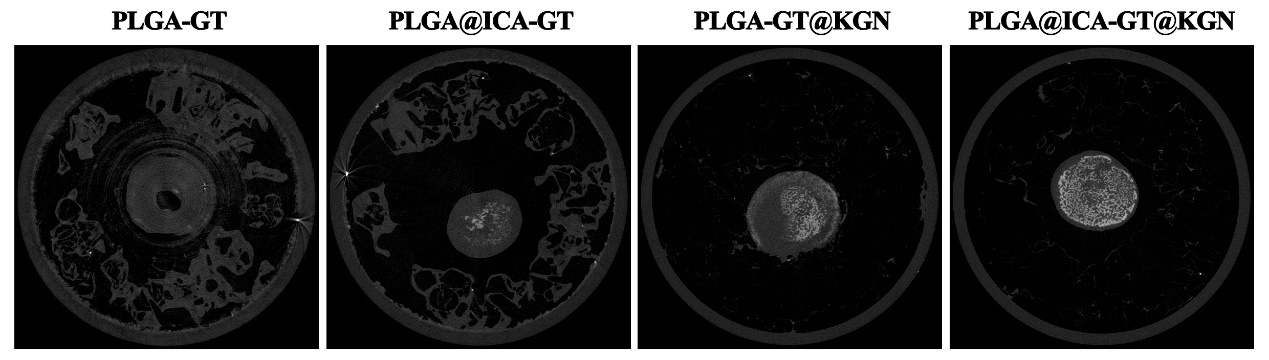


**Supplemental Figure 4.** MicroCT images for repaired grafts in PLGA-GT, PLGA@ICA-GT, PLGA-GT@KGN, and PLGA@ICA-GT@KGN groups at 2 weeks.

**
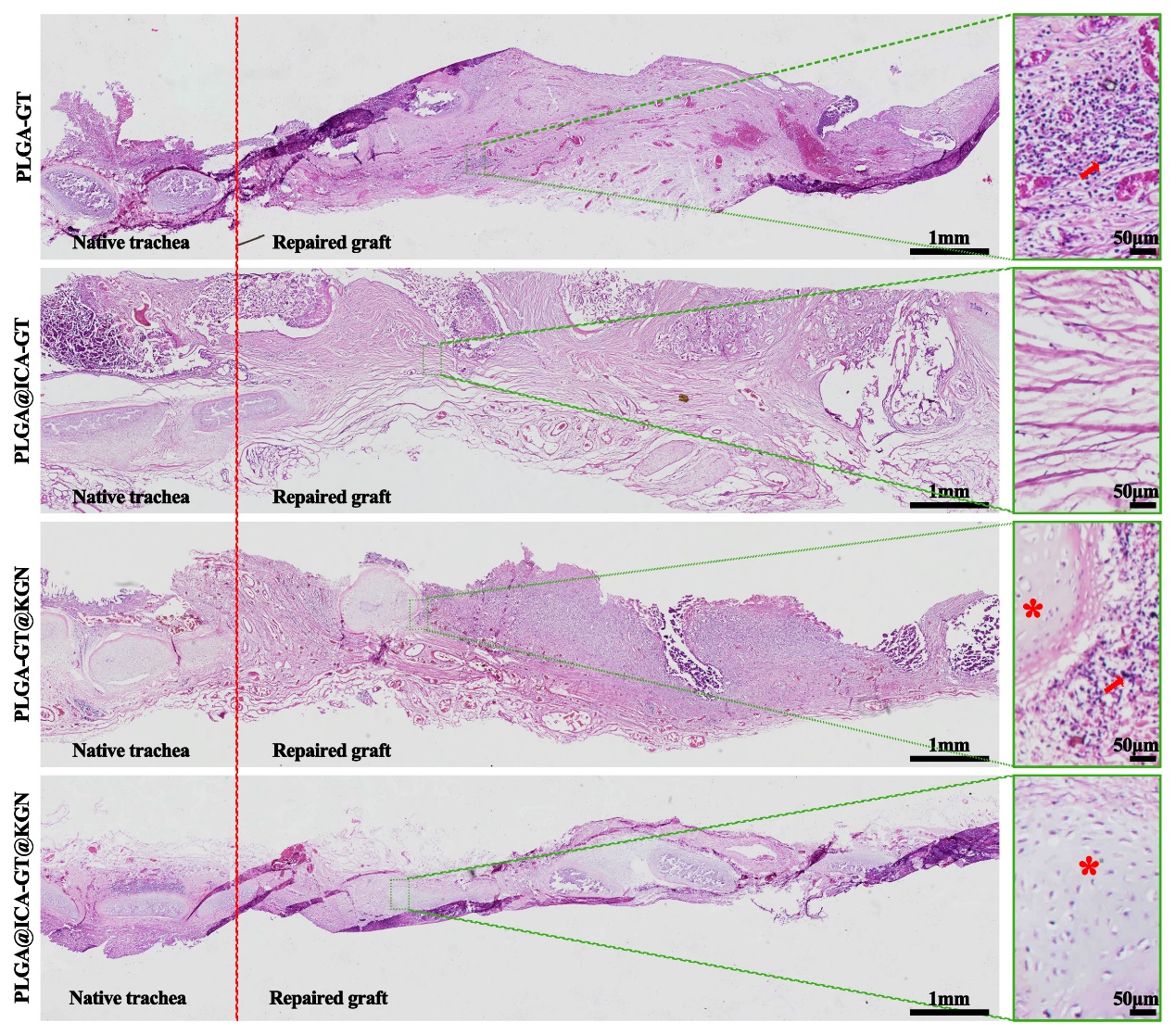
**

**Supplemental Figure 5.** HE staining for repaired grafts in PLGA-GT, PLGA@ICA-GT, PLGA-GT@KGN, and PLGA@ICA-GT@KGN groups at 2 weeks. The red dotted line marks the border between native trachea and repaired graft. The right panels are the magnified images in the left panel. The "*" mark neocartilage. The red arrows mark inflammatory cells.
